# CASC8 activates the pentose phosphate pathway to inhibit disulfidptosis in pancreatic ductal adenocarcinoma though the c-Myc-GLUT1 axis

**DOI:** 10.1186/s13046-025-03295-w

**Published:** 2025-01-27

**Authors:** Hong-Fei Yao, Jieqiong Ge, Jiahao Chen, Xiaoyan Tang, Chunjing Li, Xiao Hu, Abousalam Abdoulkader Ahmed, Yunlong Pu, Guihua Zhou, Tongyi Zhang, Zhiwei Cai, Chongyi Jiang

**Affiliations:** 1 Department of Hepato-Biliary-Pancreatic Surgery, General Surgery, Huadong Hospital, Fudan University, Shanghai, 200040 PR China; 2 Department of Nursing, Huadong Hospital, Fudan University, Shanghai, 200040 PR China

**Keywords:** Pancreatic ductal adenocarcinoma, Disulfidptosis, Pentose phosphate pathway, Cancer susceptibility 8

## Abstract

**Purpose:**

Glucose starvation induces the accumulation of disulfides and F-actin collapse in cells with high expression of SLC7A11, a phenomenon termed disulfidptosis. This study aimed to confirm the existence of disulfidptosis in pancreatic ductal adenocarcinoma (PDAC) and elucidate the role of Cancer Susceptibility 8 (CASC8) in this process.

**Methods:**

The existence of disulfidptosis in PDAC was assessed using flow cytometry and F-actin staining. CASC8 expression and its clinical correlations were analyzed using data from The Cancer Genome Atlas (TCGA) and further verified by chromogenic in situ hybridization assay in PDAC tissues. Cells with CASC8 knockdown and overexpression were subjected to cell viability, EdU, transwell assays, and used to establish subcutaneous and orthotopic tumor models. Disulfidptosis was detected by flow cytometry and immunofluorescence assays. RNA sequencing and metabolomics analysis were performed to determine the metabolic pathways which were significantly affected after CASC8 knockdown. We detected the glucose consumption and the NADP^+^/NADPH ratio to investigate alterations in metabolic profiles. RNA immunoprecipitation combined with fluorescence in situ hybridization assay was used to identify protein-RNA interactions. Protein stability, western blotting and quantitative real-time PCR assays were performed to reveal potential molecular mechanism.

**Results:**

Disulfidptosis was observed in PDAC and could be significantly rescued by disulfidptosis inhibitors. CASC8 expression was higher in PDAC samples compared to normal pancreatic tissue. High CASC8 expression correlated with a poor prognosis for patients with PDAC and contributed to cancer progression in vitro and in vivo. Furthermore, CASC8 was associated with disulfidptosis resistance under glucose starvation conditions in PDAC. Mechanistically, CASC8 interacted with c-Myc to enhance the stability of c-Myc protein, leading to the activation of the pentose phosphate pathway, a reduction of the NADP^+^/NADPH ratio and ultimately inhibiting disulfidptosis under glucose starvation conditions.

**Conclusions:**

This study provides evidence for the existence of disulfidptosis in PDAC and reveals the upregulation of CASC8 in this malignancy. Furthermore, we demonstrate that CASC8 acts as a crucial regulator of the pentose phosphate pathway and disulfidptosis, thereby promoting PDAC progression.

**Supplementary Information:**

The online version contains supplementary material available at 10.1186/s13046-025-03295-w.

## Introduction

Pancreatic ductal adenocarcinoma (PDAC) is a highly aggressive digestive malignancy. Despite accounting for only 3% of all cancers, it represents a significant clinical challenge, ranking as the third leading cause of cancer-related death in the United States [1, 2]. Therefore, developing and integrating novel targeted therapies with standard treatment regimens represent a promising strategy to improve patient outcomes by extending survival and enhancing quality of life [3–5]. In recent years, research has shown that programmed cell death (PCD), such as ferroptosis, necroptosis, and pyroptosis, plays an important role in the development and progression of PDAC under stress conditions [6–9]. Cancer cells can evade the host's immune surveillance and treatment interventions by suppressing the PCD mechanisms, thereby promoting their own proliferation and survival [10–12]. In this context, exploring the molecular mechanisms that regulate PCD provides new ideas for the treatment of PDAC.

Disulfidptosis represents a specific form of metabolism-related PCD, occurring when intracellular cystine levels become overloaded under glucose starvation conditions [13, 14]. When intracellular cystine accumulates excessively, it leads to abnormal disulfide bonds between actin cytoskeletal proteins, resulting in the collapse of the actin network and cell death. Research shows that cancer cells can respond to metabolic stress in the microenvironment through metabolic reprogramming and the regulation of disulfidptosis [15]. By employing this strategy, cancer cells are still able to acquire not only sufficient energy but also biosynthetic precursors to support their survival and proliferation [16–20]. Given this, targeting the disulfidptosis processes to promote cell death in cancer cells has emerged as a promising approach for precision therapy. However, the role and molecular regulatory mechanisms of disulfidptosis in the tumorigenesis of PDAC remain poorly understood.

By investigating the expression of disulfidptosis-related genes in PDAC, we identified disulfidptosis-related lncRNAs and revealed elevated CASC8 expression in PDAC tissues. The functions of lncRNA CASC8 in normal tissues and developmental growth remain unclear, and current research primarily focuses on its regulatory role in tumor progression, its potential as a biomarker, and its candidacy as a therapeutic target. Several studies indicate that CASC8 can influence the proliferation, invasion, and metastasis abilities of cancer cells and is also associated with patient prognosis and treatment response in lung cancer and esophageal cancer [21, 22]. A study on pancreatic cancer has demonstrated that CASC8 may serve as an effective prognostic marker and can indicate the proportion of immune infiltration in high-risk tumors [23]. Nevertheless, the functions and underlying molecular mechanisms of this lncRNA on the metabolism and disulfidptosis in PDAC have yet to be fully elucidated.

In this study, we provided hitherto undocumented evidence for disulfidptosis involvement in PDAC cells. Besides, we uncovered a tight correlation of upregulated CASC8 expression with PDAC malignancy. Modulation of CASC8 expression, achieved through either knockdown or overexpression, disrupted the pentose phosphate pathway (PPP) and altered c-Myc expression, ultimately triggering diverse cellular responses in PDAC cells under glucose starvation conditions. These findings shed light on the potential mechanisms underlying disulfidptosis and suggest CASC8 as a promising novel therapeutic target for PDAC.

## Material and methods

### Data collection

RNA sequencing data and corresponding clinical information were obtained from The Cancer Genome Atlas (TCGA) data portal (https://portal.gdc.cancer.gov/repository). The clinical data included age, gender, disease stage, tumor grade, overall survival time, and vital status (alive/deceased). Samples with missing clinical data were excluded from the analysis. Additionally, normal pancreas control samples were procured from the Genotype-Tissue Expression (GTEx) Project pancreas dataset (https://www.gtexportal.org/). A total of 178 pancreatic cancer samples were collected from TCGA, and 167 normal pancreas samples were obtained from GTEx.

### Identification of disulfidptosis-related lncRNAs in pancreatic cancer

Disulfidptosis-related genes (DRGs) were curated from the existing literature. To identify the disulfidptosis-related lncRNAs (DRLs), Pearson correlation analysis was performed. A stringent filtering criterion was employed, selecting lncRNAs with an absolute correlation coefficient (|corFilter|) greater than or equal to 0.3 and a *P*-value less than or equal to 0.001. This analysis identified a total of 173 DRLs in pancreatic cancer.

### Construction and validation of the DRL signature

A total of 178 pancreatic cancer samples obtained from TCGA were randomly divided into training and testing groups. The prognostic value of DRLs was evaluated using univariate Cox regression analysis within the training group. DRLs with statistically significant associations (*P* < 0.05) were then selected for further analysis using least absolute shrinkage and selection operator (LASSO) Cox regression and multivariate Cox regression analysis. Finally, a risk signature based on the identified DRLs was constructed in the training group. The risk scores of samples were calculated as follows:

$$\text{Risk score }=\Sigma [\text{Exp }(\text{lncRNA}) \times \text{ coef }(\text{lncRNA})]$$

where Exp denotes the expression level of lncRNAs and coef represents the corresponding coefficient for each lncRNA. Following the calculation, samples in both the training and testing groups were stratified into high- and low-risk groups based on the median risk score.

### Prognostic analysis of the risk signature

To investigate the difference in survival time between the high- and low-risk groups, we employed the 'survmine' and 'survival' R packages. We further assessed the predictive performance of the risk signature by generating receiver operating characteristic (ROC) curves and evaluating the area under the ROC curve (AUC) using the 'timeROC' R package. Univariate and multivariate Cox proportional hazards models were utilized to perform independent prognostic analysis.

### Cell culture and reagents

Human PDAC cell lines HPNE, AsPC-1, MIA PaCa-2, SW-1990, Patu8988, and PANC-1 were obtained from the Cell Bank of the Chinese Academy of Sciences (Shanghai, China). MIA PaCa-2, SW-1990, Patu8988, and PANC-1 were cultured in Dulbecco's modified Eagle's medium (DMEM) supplemented with 10% fetal bovine serum (FBS) and 1% penicillin/streptomycin (P/S). HPNE and AsPC-1 cells were cultured in Roswell Park Memorial Institute (RPMI) 1640 culture medium supplemented with 10% FBS and 1% P/S. These cells were maintained at 37 °C and 5% CO_2_ in a humidified incubator.

### Cell transfection

For the cell transfection assay, cells were seeded in 6-well plates and allowed to adhere overnight. Subsequently, each well was transfected with a combination of siRNA targeting CASC8 and the transfection reagent Lipo2000 (ThermoFisher, USA) according to the manufacturer's instructions. Following a 48-h incubation period, the cells were harvested for further experimentation. The knockdown efficiency of the siRNA was verified using quantitative real-time PCR (qRT-PCR). The siRNA sequences employed were as follows: si-CASC8-1, 5′‐GGACCAGGAGCACUAGUTT‐3′, si-CASC8-2, 5′‐GCAUGCAGCGAAUGCUCTT‐3′.

### Knockdown and overexpression assay

The lentivirus against CASC8 was purchased from Tsingke (Beijing, China), and the sequences targeting CASC8 were: sh‐1, 5′‐GCAAGAAGAGAGACCTTCATA‐3′, sh‐2, 5′‐CAGCTTCTGAGCATCCGAATA‐3′. For the CASC8 and c-Myc overexpression assay, the NR_117100.1 and NM_002467.6 sequences were utilized. The Ubi-MCS-SV40-PURO and pLKO.1-LUC-PURO vectors were employed for CASC8 overexpression and knockdown experiments, while the SV40 vector was used for c-Myc overexpression. For lentiviral transduction, cells were cultured in a 6-well plate, followed by the addition of lentiviral suspensions. 48 h post-transduction, the culture medium was supplemented with 5 µg/mL puromycin to facilitate the selection of stable cell lines.

### Cell counting kit-8

For the cell proliferation assay, cells were seeded into 96-well plates at a defined density. Subsequently, a solution of CCK-8 reagent (CCK-8, ShareBio, China) diluted to 10% in serum-free medium was added to each well at predetermined time points (0, 1, 2, 3, and 4 days). Following incubation for 1 h, the absorbance of the culture medium was measured at 450 nm using a Power Wave XS microplate reader (BIO-TEK). The resulting data were used to generate proliferation curves with GraphPad Prism 8.0 software.

### Colony formation assays

For the colony formation assay, 3000 cells were plated onto 6-well plates. Following a two-week incubation period, colonies were fixed with a 4% paraformaldehyde fixation solution. Subsequently, colonies were stained with 0.5% (w/v) crystal violet and quantified using ImageJ software.

### EdU (5‐ethynyl‐2′‐deoxyuridine)

For the EdU assay, cells were seeded onto chambered coverslips (Ibidi, 80826). After a 24-h incubation period, the medium was supplemented with EdU working solution (ShareBio, China, SB-C6015) for 2 h at 37 °C in a 5% CO₂ environment. Subsequently, the chambers were fixed with 4% paraformaldehyde fix solution for approximately 15 min at room temperature. Following the manufacturer's instructions, the EdU-positive cells were visualized and captured using a confocal microscope (Leica, Germany). In parallel, flow cytometry was employed to quantify the proportion of EdU-positive cells. The EdU Flow Cytometry Assay Kit (ShareBio, China, SB-C6020) was used according to the manufacturer's instructions.

### Quantitative real‐time PCR

Total RNA was isolated from cells using TRIzol reagent (Takara Bio, China). The isolated RNA was then reverse-transcribed into cDNA using the PrimeScript RT Master Mix reagent (Takara Bio, China), following the manufacturer's instructions. Quantitative real-time PCR (qRT-PCR) was performed using Universal SYBR Green qPCR Premix (ShareBio, China) on a 7500 real-time PCR system (Applied Biosystems, USA). The cycling conditions recommended by the manufacturer were employed. Relative mRNA expression levels were calculated using the 2^−ΔΔCT^ method. 18S ribosomal RNA was chosen as the internal reference gene for normalization. The primer sequences are listed in the supplementary table.

### Western blotting

Cells were lysed using RIPA buffer (Thermo Fisher Scientific, USA) to extract total protein. The proteins were separated by sodium dodecyl sulfate–polyacrylamide gel electrophoresis (SDS-PAGE) and transferred onto nitrocellulose membranes. Following the transfer, the membranes were blocked with 5% non-fat dry milk in Tris-buffered saline with 0.1% Tween-20 (TBST) for approximately 1 h at room temperature. After blocking, the membranes were incubated with primary antibodies diluted in TBST containing bovine serum albumin (BSA) or non-fat dry milk to prevent non-specific binding. Specific primary antibodies used were: β-actin (Abcam, ab8227, 1:5000), GLUT1 (Proteintech, 21,829–1-AP, 1:1000), ENO1 (Proteintech, 11,204–1-AP, 1:2000), GPI (Proteintech, 15,171–1-AP, 1:1000), LDHA (Proteintech, 19,987–1-AP, 1:2000), HK2 (Proteintech, 66,974–1-Ig, 1:5000), and c-Myc (Proteintech, 10,828–1-AP, 1:2000). Following incubation with primary antibodies, the membranes were washed in TBST and then incubated with horseradish peroxidase (HRP)-conjugated secondary antibodies (goat anti-mouse IgG, ShareBio, 1:5000; goat anti-rabbit IgG, ShareBio, 1:5000) in TBST containing BSA or non-fat dry milk for 1 h at room temperature. Finally, protein bands were visualized using enhanced chemiluminescent (ECL) reagents (ShareBio, China).

### Transwell chamber assays

For transwell migration assay, 4 × 10^4^ cells were resuspended in a serum-free medium and seeded onto the upper chamber of the transwell insert. The lower chamber was filled with a medium containing 10% FBS, which served as a chemoattractant. Following a 48-h incubation period, the transwell chambers were fixed with 4% paraformaldehyde solution for 15 min at room temperature to preserve cell morphology. Subsequently, the cells were stained with 0.5% crystal violet solution to visualize migrated cells. Finally, the number of migrated cells was quantified using ImageJ software.

### Flow cytometry

For flow cytometry assay, 2 × 10^5^ cells were seeded into 6-well plates and cultured in a complete medium either containing or lacking glucose for 12 h. To investigate the effect of disulfide stress on cell death, Dithiothreitol (DTT) and tris-(2-carboxyethyl)-phosphine (TCEP), two reagents known to prevent disulfide stress, were added to specific cultures. Following treatment, cells were collected and stained with propidium iodide (PI), a fluorescent dye that can only enter dead cells with compromised membranes. Dead cells were then identified and quantified using a flow cytometer (BD AccuriTM C6 Plus cell analyzer, BD Biosciences, USA). Flow cytometry data was analyzed using FlowJo 10.4 software to determine the percentage of PI-positive cells, representing the proportion of dead cells in the population.

### Fluorescent staining of actin filaments

Cells were seeded onto chambered coverslips (Ibidi, 80,826) and adhered overnight. Subsequently, the cells were fixed with 4% paraformaldehyde for 15 min at room temperature to preserve their morphology. After fixation, the cells were permeabilized with 0.5% Triton X-100 for 5 min at room temperature to facilitate antibody access to intracellular antigens. The cells were then washed three times with phosphate-buffered saline (PBS) to remove residual fixative and permeabilization buffer. Following washing, the cells were stained with 100 nM Actin-stain 555 phalloidin (Thermo Fisher Scientific, USA) for approximately 30 min to visualize the F-actin cytoskeleton. After incubation, the cells were washed three times with PBS to remove unbound phalloidin. Nuclei were then counterstained with DAPI (Thermo Fisher Scientific, USA) for 5 min to visualize DNA. Finally, the coverslips were mounted onto slides, and images were captured using a confocal microscope (Leica, Germany).

### Glucose consumption assay

PDAC cells were seeded into 6-well plates and cultured for approximately 24 h. Following incubation, the culture medium was collected, and the remaining glucose concentration was measured using the BioVision Glucose Uptake Assay Kit (BioVision, K676) according to the manufacturer's instructions. The amount of glucose uptake by the cells was then calculated by subtracting the remaining glucose concentration in the culture medium from the initial glucose concentration (typically provided by the manufacturer). To normalize for cell number, the glucose uptake values were normalized to the total protein content of the cells, which was determined using a bicinchoninic acid (BCA) assay.

### NADP^+^ and NADPH measurement

The intracellular NADP^+^ and NADPH levels were determined using the NADP^+^/NADPH Assay Kit (Beyotime, China, S1079), following the manufacturer's instructions. Briefly, PDAC cells were seeded into 6-well plates and cultured for approximately 24 h. The culture medium was then discarded, and the cells were lysed using the NADP^+^/NADPH extraction reagent provided in the kit. The NADP^+^/NADPH ratio in the cell lysates was then measured according to the kit instructions.

### Human PDAC sample collection

Primary PDAC tissues were collected from patients diagnosed with PDAC who were undergoing treatment at Huadong Hospital, Fudan University. All patients provided written informed consent prior to tissue collection in accordance with ethical guidelines. Pathological data associated with the tissue samples were obtained from the Pathology Department.

### Subcutaneous and orthotopic xenograft models

Six- to eight-week-old male athymic nude (nu/nu) mice were used to establish subcutaneous and orthotopic xenograft tumor models. For the subcutaneous xenograft model, PDAC cells were resuspended in 100 μL of sterile PBS and subcutaneously injected into the flank region of the mice. After four weeks of inoculation, tumors were surgically resected, and their weights were measured. For the orthotopic xenograft model, mice were anesthetized using isoflurane according to institutional guidelines. Subsequently, PDAC cells were resuspended in 50 μL of sterile PBS and directly injected into the pancreas of the mice. Following four weeks of inoculation, the mice were euthanized, and tumors were excised, fixed in a 4% paraformaldehyde solution, weighed, and photographed.

### Immunofluorescence (IF) assay

For immunofluorescence analysis of patient tumor tissues, samples were fixed in 4% paraformaldehyde solution, embedded in paraffin wax, and subsequently processed for IF staining. The primary antibody used was specific for CK19 (Servicebio, GB15198-100, 1:500). DAPI (Servicebio, G1012) was used for a 5-min counterstain of the nuclei to visualize DNA. For IF analysis of PDAC cells, cells were seeded onto chambered coverslips (Ibidi, 80,826) and cultured for 24 h. Subsequently, the cells were fixed with 4% paraformaldehyde solution for 10 min at room temperature to preserve their morphology. After fixation, the cells were permeabilized with 0.5% Triton X-100 for 5 min to facilitate antibody access to intracellular antigens. Following permeabilization, the cells were blocked with 5% BSA for 1 h at room temperature to prevent non-specific antibody binding. The cells were then incubated with primary antibodies specific for GLUT1 (Proteintech, 21,829–1-AP, 1:200) and c-Myc (Proteintech, 10,828–1-AP, 1:200) overnight at 4 °C. Following incubation with primary antibodies, the cells were washed and incubated with a secondary antibody (goat anti-rabbit IgG) for 1 h at room temperature. Finally, the cells were stained with DAPI (Servicebio, G1012) for 5 min to visualize nuclei. Digital images were captured using confocal microscopes (Leica, Germany).

### Immunohistochemistry (IHC) and TUNEL assay

For IHC analysis, xenograft tumors were fixed in a 4% paraformaldehyde solution, embedded in paraffin wax, and subsequently processed for IHC staining. Primary antibodies specific for c-Myc (Servicebio, GB13076-50, 1:200) and Ki-67 (Servicebio, GB121141-100, 1:300) were used to stain the tissue sections. The Terminal deoxynucleotidyl transferase dUTP nick-end labeling (TUNEL) assay was performed to identify apoptotic cells in paraffin sections of mouse xenografts. A TUNEL kit (Servicebio, GB1507-50 T) was used according to the manufacturer's instructions.

### Chromogenic in situ hybridization (CISH)

CISH was performed on tissue sections following established protocols. Briefly, the sections underwent deparaffinization and dehydration to remove embedding media. This was followed by antigen retrieval, a process that enhances the accessibility of target molecules for probe binding. Subsequently, the sections were treated with protease K, an enzyme that digests proteins and facilitates probe penetration. After pre-hybridization to create optimal conditions for probe binding, the sections were hybridized with a solution containing specific DNA probes complementary to the target RNA sequence. Following hybridization, the sections were washed to remove unbound probes and then blocked with rabbit serum to prevent non-specific antibody binding. The target RNA was then visualized using a colorimetric detection system. Finally, the sections were counterstained with a nuclear stain, sealed with a coverslip, and imaged using a microscope.

### RNA sequencing

Total RNA was extracted from MIA PaCa-2 cells in both the control group and the CASC8 knockdown group (n = 3) using the TRIzol reagent (Invitrogen, CA, USA) according to the manufacturer’s instructions. Then the libraries were constructed using VAHTS Universal V6 RNA-seq Library Prep Kit according to the manufacturer’s instructions. The libraries were sequenced on a llumina Novaseq 6000 platform and 150 bp paired-end reads were generated. Raw reads were firstly processed using fastp (v0.22.0) and the low-quality reads were removed to obtain the clean reads. The clean reads were mapped to the reference genome using HISAT2 (v2.1.0). FPKM of each gene was calculated and the read counts of each gene were obtained by featureCounts. The transcriptome sequencing and analysis were conducted by OBiO Technology Corp., Ltd. (Shanghai, China).

### RNA immunoprecipitation

The antibodies utilized included anti-c-Myc (Proteintech, 10,828–1-AP) and anti-IgG (Abclone, AC005). Briefly, cell samples were harvested from a 15 cm culture plate when the cell confluence reached 80–90% using 250 μL of cell lysis buffer. The A/G magnetic beads were washed twice and incubated with anti-IgG and anti-c-Myc at room temperature for 1 h. A 20 μL sample from each lysate was collected and stored at -80 °C as RNA input. Subsequently, 100 μL of lysate was added to the prepared anti-IgG beads, while an additional 100 μL of lysate was added to the anti-c-Myc beads. These beads were incubated overnight at 4 °C with rotation. The magnetic beads were then washed six times with 1 mL of wash buffer. The resuspended beads were collected for RNA isolation using phenol: chloroform: isoamyl alcohol (125:24:1) reagent. Each RNA sample was reverse-transcribed for subsequent qPCR analysis.

### Metabolism analysis

The MIA PaCa-2 CASC8 knockdown cells, SW-1990 CASC8 overexpression cells, and their respective control cells (n = 3) were washed with PBS under 37 °C for three times and the PBS was removed. The sample was proceeded with trypsinization for 2 min, then serum medium was added to stop the reaction. The cells were collected into a new centrifuge tube, and centrifuged at 14000 g for 5 min to remove supernatant. The cell pellets were washed with PBS under 4 °C for three times, and centrifuged at 14000 g for 5 min to remove the PBS. Then the cell pellets were quickly frozen in liquid nitrogen and immediately proceeded with extraction or store the material at -80 °C. 1000 μL methanol/acetonitrile/H2O (2:2:1, v/v/v) were added to homogenized solution for metabolite extraction. The mixture was centrifuged for 15 min (14000 g, 4 °C). The supernatant was dried in a vacuum centrifuge. For LC–MS analysis, the samples were re-dissolved in 100 μL acetonitrile/water (1:1, v/v) solvent. Analyses were performed using an UHPLC (1290 Infinity LC, Agilent Technologies) coupled to a quadrupole time-of-flight (AB Sciex TripleTOF 6600) in OBiO Technology Corp., Ltd. (Shanghai, China).

### Statistical analysis

The bioinformatics analysis was performed using R software (version 4.0.2) for numerical data analysis and visualization. Data were presented as mean ± standard deviation. Statistical significance between groups was determined using appropriate tests, including Chi-square, Student's t-test, or ANOVA. For survival curve analysis, the Log-rank test was used. All in vitro experiments were repeated at least three times, and a *P*-value threshold of 0.05 was used to define statistical significance.

## Results

### Disulfidptosis and the expression patterns of DRGs identified in PDAC

**Fig. 1:**
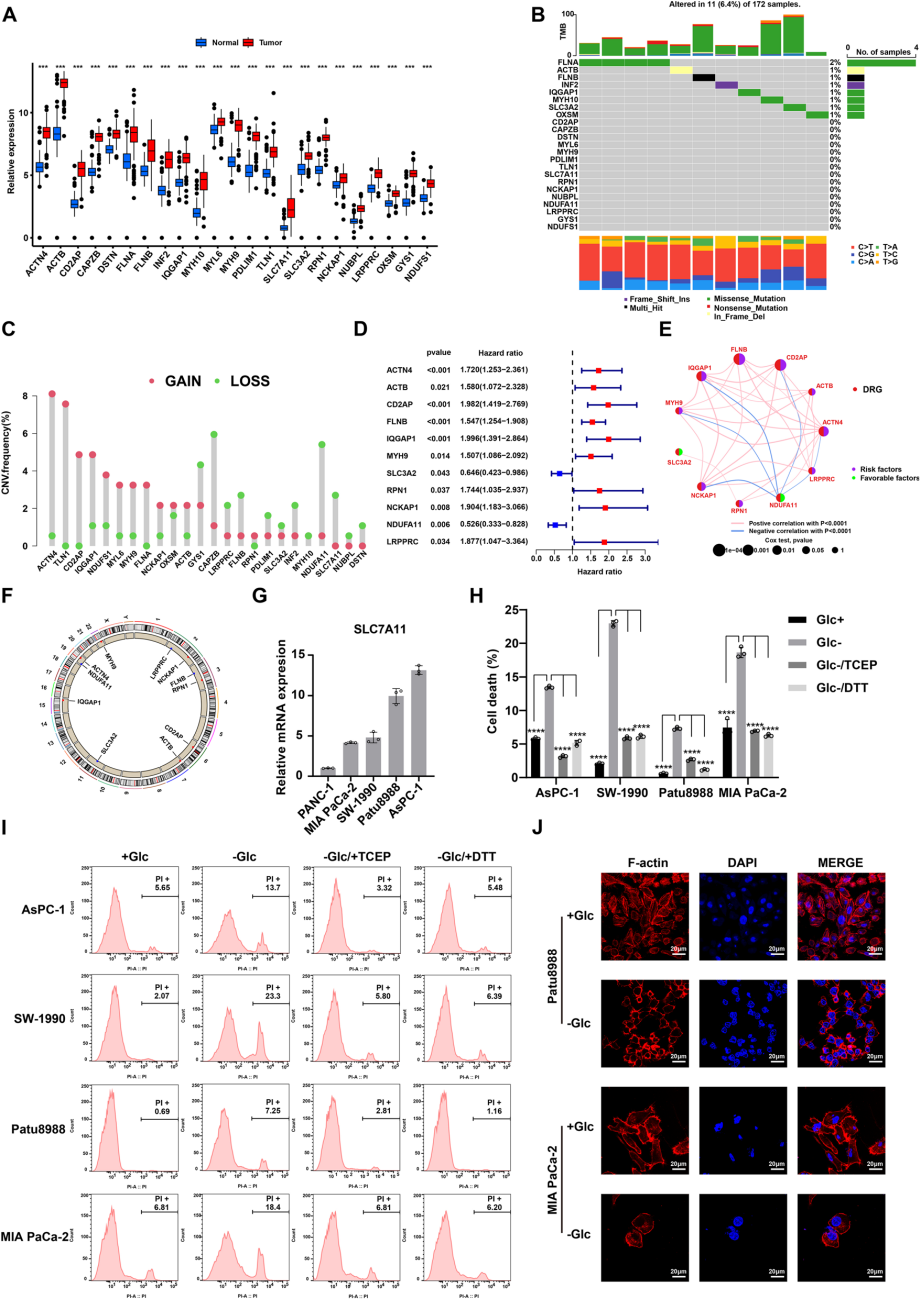
The presence of disulfidptosis in PDAC. **A** Expression levels of DRGs in normal pancreas and pancreatic cancer. The expression data was downloaded from TCGA-PAAD and GTEx-Pancreas datasets. **B** Mutational information of DRGs in pancreatic cancer samples from the TCGA database. **C** Copy number variation of DRGs in pancreatic cancer. **D** Forest plot of DRGs from the results of univariate Cox regression analysis. **E** Correlation and prognostic values of DRGs in pancreatic cancer. **F** Location of DRGs in chromosomes. **G** The relative expression level of SLC7A11 in PDAC cell lines. **H**, **I** Proportion of PI-positive dead cells after cultured in a glucose-free medium for 12 h and treated with 0.5 mM DTT or 1 mM TCEP in PDAC cells. The results were analyzed by one-way ANOVA. **J** Fluorescence staining of F-actin with phalloidin in PDAC cells under glucose starvation for 12 h. Scale bar = 20 μm. ****P < 0.0001; ***P < 0.001; **P < 0.01; *P < 0.05

In previous studies, 24 DRGs were summarized through exact experiments. To further investigate the expression patterns of DRGs in PDAC and normal pancreas tissues, we downloaded and analyzed data from The Cancer Genome Atlas (TCGA-PAAD) and GTEx projects. As shown in Fig. 1A, most DRGs exhibited significantly higher expression in PDAC samples compared to normal controls. Interestingly, these DRGs displayed an extremely low mutation rate within the TCGA-PAAD cohort (Fig. 1B). Analysis of copy number variation (CNV) frequencies revealed ACTN4 and TLN1 as the top two genes with CNV amplifications, while CAPZB and NUDFA11 showed CNV deletions (Fig. 1C). Additionally, univariate Cox regression analysis identified two favorable and nine unfavorable prognostic factors associated with PDAC (Fig. 1D-E). The chromosomal locations of these eleven genes are presented in Fig. 1F.

To investigate the potential role of disulfidptosis in PDAC, we first evaluated SLC7A11 expression levels in five PDAC cell lines. Four cell lines exhibiting high SLC7A11 expression were subsequently selected for further analysis (Fig. 1G). Following a 12-h incubation period in a glucose-depleted medium, a significant increase in the percentage of PI-positive cells was observed (*P* < 0.001) (Fig. 1H). The specific data were as follows: With glucose: AsPC-1 = 5.65%−5.77%, SW-1990 = 1.92%−2.20%, Patu8988 = 0.52%−0.69%, and MIA PaCa-2 = 6.70%−6.81%; Without glucose: AsPC-1 = 13.30%−13.70%, SW-1990 = 22.50%−23.30%, Patu8988 = 7.13%−7.56%, and MIA PaCa-2 = 18.00%−19.50%. Notably, the addition of DTT and TCEP – known mitigators of disulfide stress and disulfidptosis – to the glucose-free medium resulted in a marked decrease in PI-positive cells (F ig. 1I). Furthermore, fluorescence staining revealed morphological changes in cells subjected to glucose starvation, including F-actin contraction and cell shrinkage (Fig. 1J). Collectively, these findings suggest a potential contribution of disulfidptosis to PDAC progression.

### Risk signature based on disulfidptosis-related lncRNAs is constructed in PDAC

**Fig. 2:**
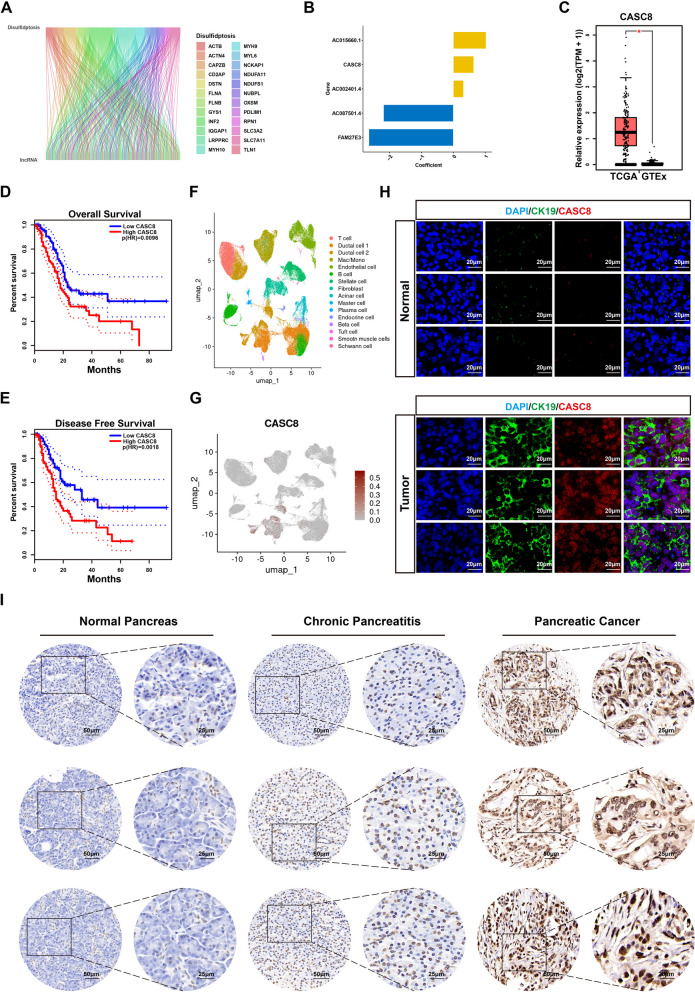
The expression level and prognostic value of CASC8 in PDAC. **A** The Sankey diagram showing the relationship between DRGs and DRLs. **B** Coefficient of five disulfidptosis-related lncRNAs in the risk signature. **C** The expression level of CASC8 in TCGA-PAAD and GTEx-Pancreas datasets. The statistical analysis was done by unpaired t-test. **D**, **E** Kaplan–Meier survival curve analysis of OS (**D**) and DFS (**E**) was conducted between the high and low CASC8 groups using data from TCGA. **F** Cell types identified by the single-cell RNA sequencing dataset CRA001160. **G** The expression level of CASC8 across various cell types identified by the dataset CRA001160. **H** Fluorescent staining of CK19 and FISH of CASC8 in normal pancreas and PDAC tissues. Scale bar = 20 μm. **I** CISH of CASC8 in normal pancreas, chronic pancreatitis, and PDAC tissues. ***P < 0.001; **P < 0.01; *P < 0.05

To identify DRLs, we employed the limma package to explore the correlation between lncRNAs and disulfidptosis-related genes in PDAC samples. Stringent criteria of |cor|≥ 0.3 and *P*-value ≤ 0.001 were used for this analysis. This approach identified a total of 173 DRLs (Fig. 2A). Subsequently, pancreatic cancer samples from TCGA were randomly divided into training and testing groups. Univariate Cox proportional hazards regression analysis revealed 54 lncRNAs significantly associated with prognosis in the training group (*P* < 0.05) (Supplementary Fig. 1A). These 54 DRLs were then subjected to LASSO regression and multivariate Cox regression analyses (Supplementary Figs. 1B-C). As a result, five key DRLs, including AC002401.4, CASC8, AC015660.1, AC087501.4, and FAM27E3, were identified and used to establish a risk signature (Fig. 2B). The risk score for each sample was calculated using the following formula: risk score = ∑ (expression level of lncRNA * coefficient), where the coefficients were: AC002401.4 × (0.320623768147482) + CASC8 × (0.640108395445088) + AC087501.4 × (-2.18492037606468) + FAM27E3 × (-2.6447642798446) + AC015660.1 × (1.02658147843575). Based on the median risk score, samples in the training, testing, and entire cohorts were categorized into high- and low-risk groups. We further compared the expression levels of these five lncRNAs between the high- and low-risk groups in the training set. As expected, the prognostic risk factors, AC002401.4, CASC8, and AC015660.1, were significantly upregulated in the high-risk group, while AC087501.4 and FAM27E3 displayed the opposite trend (Supplementary Fig. 1D). The risk score and survival status for each sample were presented in Supplementary Figs. 1E-F. Notably, the results indicated that samples in the high-risk group had a significantly higher mortality rate, which was further confirmed by Kaplan–Meier curve analysis (Supplementary Figs. 1G-H). Patients in the high-risk group exhibited a demonstrably shorter overall survival (OS) (*P* < 0.001) and progression-free survival (PFS) (*P* = 0.001). The AUC from the ROC analysis and the independent prognostic analysis suggested that the risk signature not only possessed excellent prognostic prediction ability but also served as an independent prognostic factor for PDAC patients (Supplementary Figs. 1I-K).

### Validation of the risk signature in the testing group and the entire cohort

To further assess the generalizability of the risk signature, we validated its performance in the testing group and the entire cohort. The risk score for each sample was calculated using the formula established in the training group. As anticipated, the expression patterns of the five lncRNAs and the survival outcomes in the high- and low-risk groups of the testing and entire cohorts mirrored those observed in the training group (Supplementary Fig. 2A-F). Consistent with these findings, Kaplan–Meier curve analyses revealed that patients with higher risk scores exhibited significantly poorer prognoses, as evidenced by both OS and PFS (Supplementary Fig. 2G-J). Furthermore, the AUC from ROC analyses in the testing and entire cohorts confirmed the strong predictive power of the risk signature (Supplementary Fig. 2 K-L). Finally, Cox regression analyses demonstrated that the risk signature served as an independent prognostic factor in both the testing and entire cohorts (Supplementary Fig. 2 M-P), consistent with the results observed in the training group. Collectively, these validation steps establish the robustness and generalizability of the risk signature.

### CASC8 is upregulated and correlated with poor prognosis in PDAC

To further investigate the expression pattern and prognostic value of lncRNAs within the risk signature in PDAC, we utilized the GEPIA database (http://gepia2.cancer-pku.cn/). The analysis revealed that CASC8 expression was higher in pancreatic cancer samples compared to controls (Log2FC = 1.245, adjusted *P* = 2.29E-56). Furthermore, higher CASC8 expression was significantly associated with shorter OS (*P* = 0.0096) and DFS (*P* = 0.0018) rates in patients (Supplementary Figs. 3A-L, Fig. 2C-E). To validate the expression level of CASC8 in tumor cells, we obtained a single-cell RNA sequencing dataset (scRNA-seq; CRA001160). This analysis demonstrated that CASC8 expression was predominantly localized to tumor cells (Fig. 2F-G). Fluorescence in situ hybridization (FISH) analysis of PDAC samples further corroborated these findings, revealing significantly higher CASC8 expression in tumor cells compared to normal cells (Fig. 2H). Consistent results were also obtained with the CISH assay. Notably, PDAC tissues exhibited markedly higher CASC8 expression than normal pancreatic tissues and chronic pancreatitis samples (F ig. 2I). Collectively, these data suggest that CASC8 is upregulated and may serve as a predictive indicator in PDAC progression.

### CASC8 promotes PDAC growth and migration in vitro

To elucidate the functional effects of CASC8 on PDAC progression, we initially investigated its expression levels in human PDAC cell lines compared to the non-malignant pancreatic ductal epithelial cell line HPNE. As expected, three PDAC cell lines displayed significantly higher CASC8 expression compared to HPNE cells (Supplementary Fig. 4A). To explore the potential roles of CASC8 in PDAC, we employed short interfering RNA (siRNA) for CASC8 knockdown in Patu8988 and MIA PaCa-2 cells, while SW-1990 and AsPC-1 cells were transfected with constructs for CASC8 exogenous overexpression. The effectiveness of CASC8 knockdown and overexpression was confirmed by qRT-PCR analysis (Supplementary Fig. 4B-C). In vitro experiments revealed that CASC8 knockdown significantly suppressed the proliferation of PDAC cells, as demonstrated by CCK-8 and colony formation assays (Supplementary Fig. 4D-F). Conversely, overexpression of CASC8 enhanced the proliferative capacity of PDAC cells (Supplementary Fig. 4G-I). Furthermore, the EdU assay indicated a significantly higher proportion of EdU-positive cells (actively proliferating cells) in the control cells compared to the CASC8 knockdown cells (Supplementary Fig. 4 J). Flow cytometry analysis further corroborated these findings, demonstrating a statistically greater percentage of EdU-positive cells in the CASC8 overexpression cells (Supplementary Fig. 4 K-L). Notably, CASC8 knockdown and overexpression also demonstrably modulated PDAC cell migration in vitro (Supplementary Fig. 4 M-P). Collectively, these results strongly suggest that CASC8 plays a contributory role in promoting PDAC progression in vitro.

### CASC8 inhibits disulfidptosis under glucose starvation conditions in PDAC cells in vitro

**Fig. 3:**
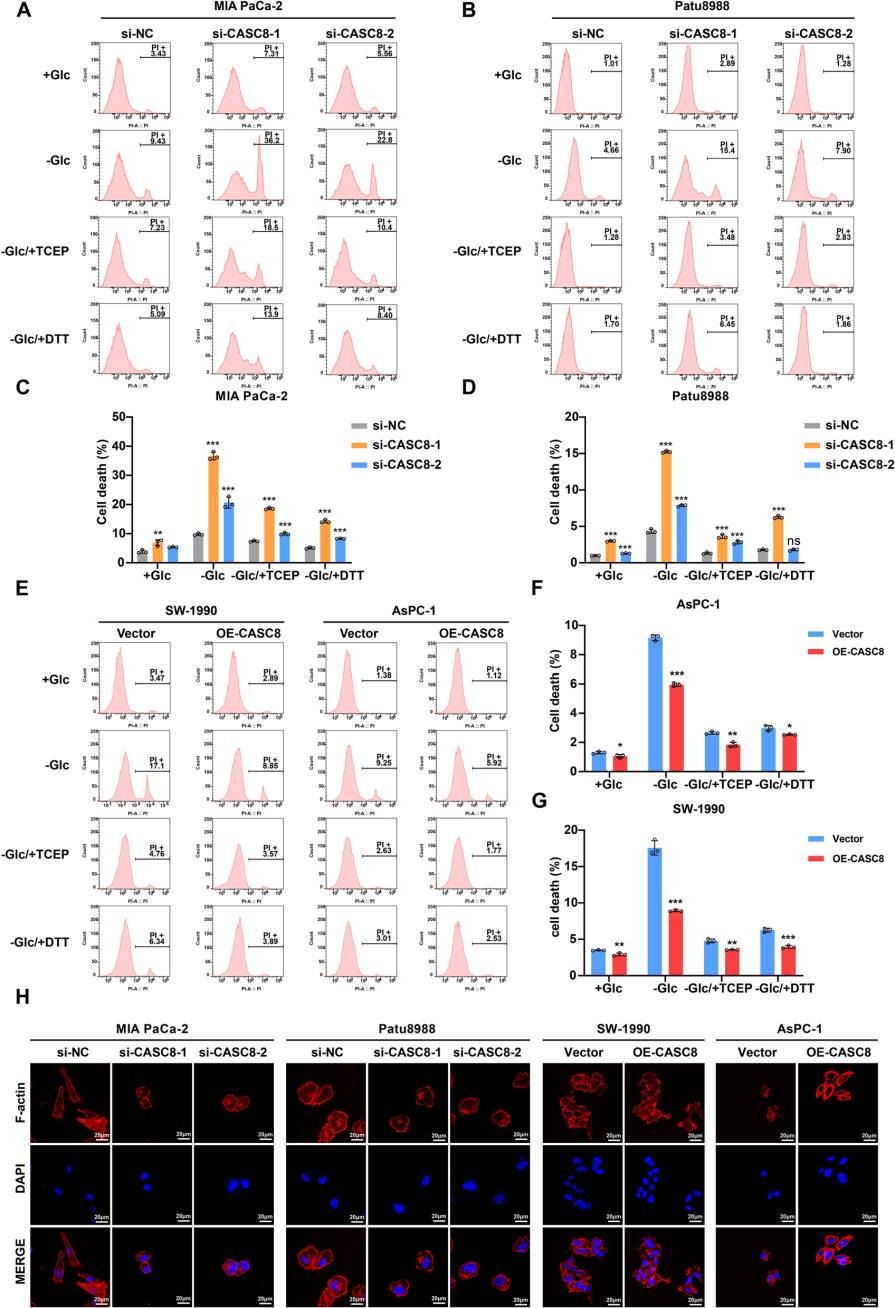
The roles of CASC8 in regulating disulfidptosis in PDAC. **A**, **B** The effects of CASC8 knockdown on the death of MIA PaCa-2 (**A**) and Patu8988 (**B**) after cultured in glucose-free medium for 12 h and treated with 0.5 mM DTT or 1 mM TCEP. **C**, **D** The results of statistical analysis of the PI-positive cells in MIA PaCa-2 (**C**) and Patu8988 (**D**). The statistical analyses were done by one-way ANOVA. **E** The effects of CASC8 overexpression on the death of SW-1990 and AsPC-1 after cultured in glucose-free medium for 12 h and treated with 0.5 mM DTT or 1 mM TCEP. **F**, **G** The results of statistical analysis of the PI-positive cells in AsPC-1 (**F**) and SW-1990 (**G**). The results were analyzed by unpaired t-test. **H** Fluorescent staining of F-actin with phalloidin in PDAC cells after CASC8 knockdown or overexpression. The cells were cultured under glucose starvation for 12 h. Scale bar = 20 μm. ***P < 0.001; **P < 0.01; *P < 0.05

Disulfidptosis, a recently discovered form of cell death triggered by metabolic stress and glucose starvation, is linked to the formation of disulfide bonds within cellular proteins. Given our identification of CASC8 as a disulfidptosis-related lncRNA, we further investigated its impact on this process in PDAC cells. Using flow cytometry to assess cell death, we observed a significant increase in the proportion of PI-positive dead cells following CASC8 knockdown under glucose starvation conditions (*P* < 0.001). The specific data were as follows: MIA PaCa-2: si-NC = 9.43%−10.20%, si-CASC8-1 = 35.60%−38.20%, si-CASC8-2 = 19.20%−22.80%; Patu8988: si-NC = 4.13%−4.66%, si-CASC8-1 = 15.10%−15.40%, si-CASC8-2 = 7.70%−7.93%. Notably, this increase was significantly reversed by the addition of the reducing agents TCEP and DTT (Fig. 3A-D). Conversely, CASC8 overexpression showed a contrasting trend, with a lower percentage of PI-positive cells observed under glucose starvation (*P* < 0.001) (Fig. 3E-G). The specific data were as follows: AsPC-1: Vector = 8.94%−9.29%, OE-CASC8 = 5.85%−6.08%; SW-1990: Vector = 16.90%−18.70%, OE-CASC8 = 8.84%−9.07%. Furthermore, fluorescent staining revealed morphological changes consistent with disulfidptosis, including F-actin contraction and cell shrinkage, in the CASC8 knockdown and vector cells compared to the control and CASC8 overexpression cells (Fig. 3H). Collectively, these data strongly suggest that CASC8 plays a prominent role in regulating disulfidptosis in PDAC cells.

### CASC8 inhibits PDAC disulfidptosis in vivo

**Fig. 4:**
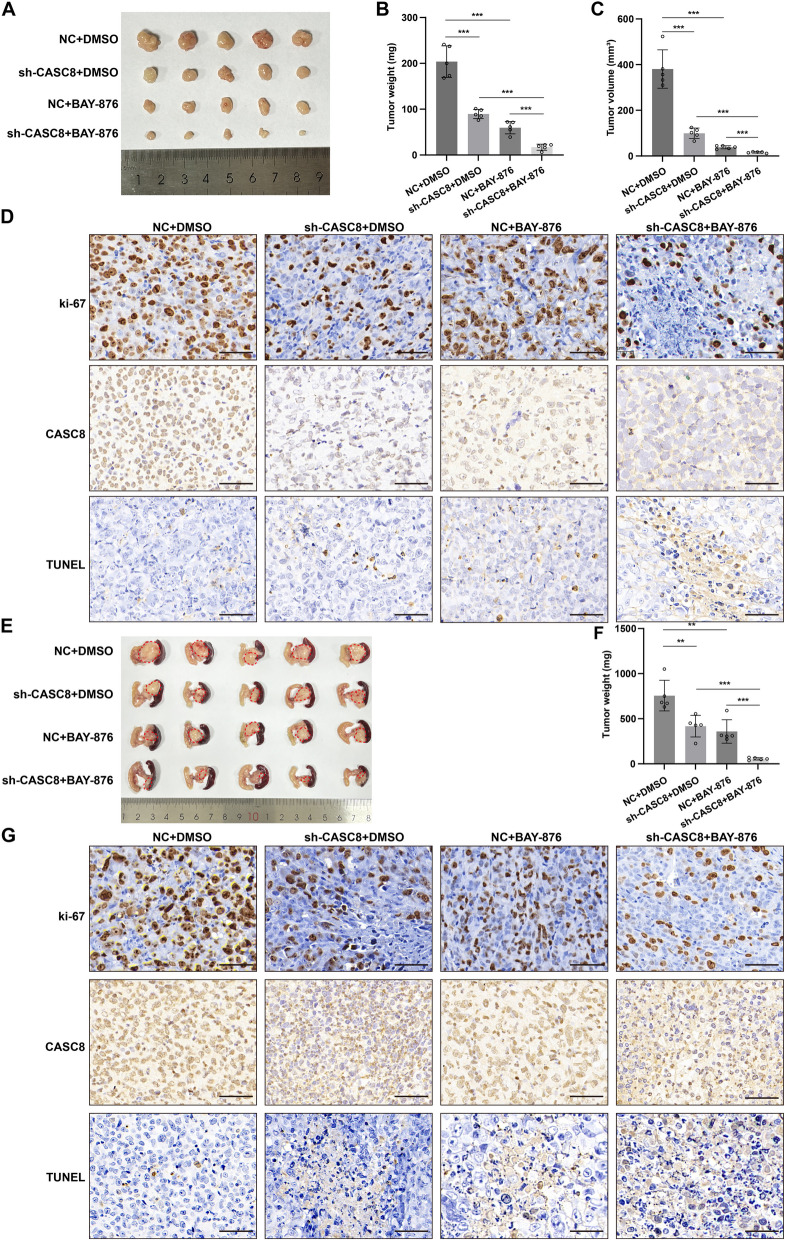
CASC8 inhibits PDAC disulfidptosis in vivo. **A** Subcutaneous xenograft tumor growth in mice inoculated with shNC, sh-CASC8 MIA PaCa-2 (n = 5 for each group). After implanting the tumor subcutaneously for 7 days, each group of mice received intraperitoneal injections of BAY-876 at a dose of 3 mg/kg in 100 μL of 40% dimethylsulfoxide in saline (vehicle) or vehicle alone every two days. **B**, **C** Statistical analysis of tumor weight and volume of subcutaneous xenograft tumors from different groups. *P*-values between the two interested groups were calculated by unpaired t-test. **D** Representative images of IHC staining for ki-67, and CISH for CASC8, and TUNEL assay in subcutaneous xenograft tumors. Scale bar = 20 μm. **E** Orthotopic xenograft tumor growth in mice inoculated with shNC, sh-CASC8 MIA PaCa-2 (n = 5 for each group). After implanting the tumor orthotopically for 7 days, each group of mice received intraperitoneal injections of BAY-876 at a dose of 3 mg/kg in 100 μL of 40% dimethylsulfoxide in saline (vehicle) or vehicle alone every two days. **F** Statistical analysis of tumor weight of orthotopic xenograft tumors from different groups. *P*-values between the two interested groups were calculated by unpaired t-test. **G** Representative images of IHC staining for ki-67, and CISH for CASC8, and TUNEL assay in orthotopic xenograft tumors. *P*-values between the two interested groups were calculated by unpaired t-test. Scale bar = 20 μm. ***P < 0.001; **P < 0.01; *P < 0.05

To investigate the in vivo effects of CASC8 on disulfidptosis, we established stable CASC8 knockdown PDAC cells using lentiviral short hairpin RNA (shRNA) constructs. Subsequently, we developed a subcutaneous xenograft tumor model. Compared the xenografts from the control group, five mice injected with sh-CASC8 cells exhibited a significantly lower tumor burden (*P* < 0.001) (Fig. 4A-C). Notably, treatment with 3 mg/kg of BAY-876, a GLUT1 inhibitor that mimics glucose starvation, produced similar anti-tumor effects, suggesting a potential link between CASC8 and glucose metabolism in vivo (*P* < 0.001). Paraffin-embedded xenograft tumors were subjected to IHC staining for Ki-67, and CISH for CASC8, along with TUNEL assays (Fig. 4D). To validate these findings, we further employed an orthotopic xenograft model, where tumor cells were implanted into the pancreas of mice. Consistent with the results from the subcutaneous model, five mice in the negative control group developed significantly larger and heavier tumors compared to the sh-CASC8 group (*P* = 0.0065). Importantly, treatment with 3 mg/kg of BAY-876 in the orthotopic model maintained this observed difference in tumor size (*P* < 0.001) (Fig. 4E-F). Concurrently, the IHC results also indicated a stronger proliferation capacity in the control group than in the sh-CASC8 group (Fig. 4G). Overall, these in vivo findings strongly suggest that CASC8 can inhibit disulfidptosis in PDAC cells, potentially by modulating glucose metabolism.

### CASC8 is associated with the pentose phosphate pathway in PDAC cells

**Fig. 5:**
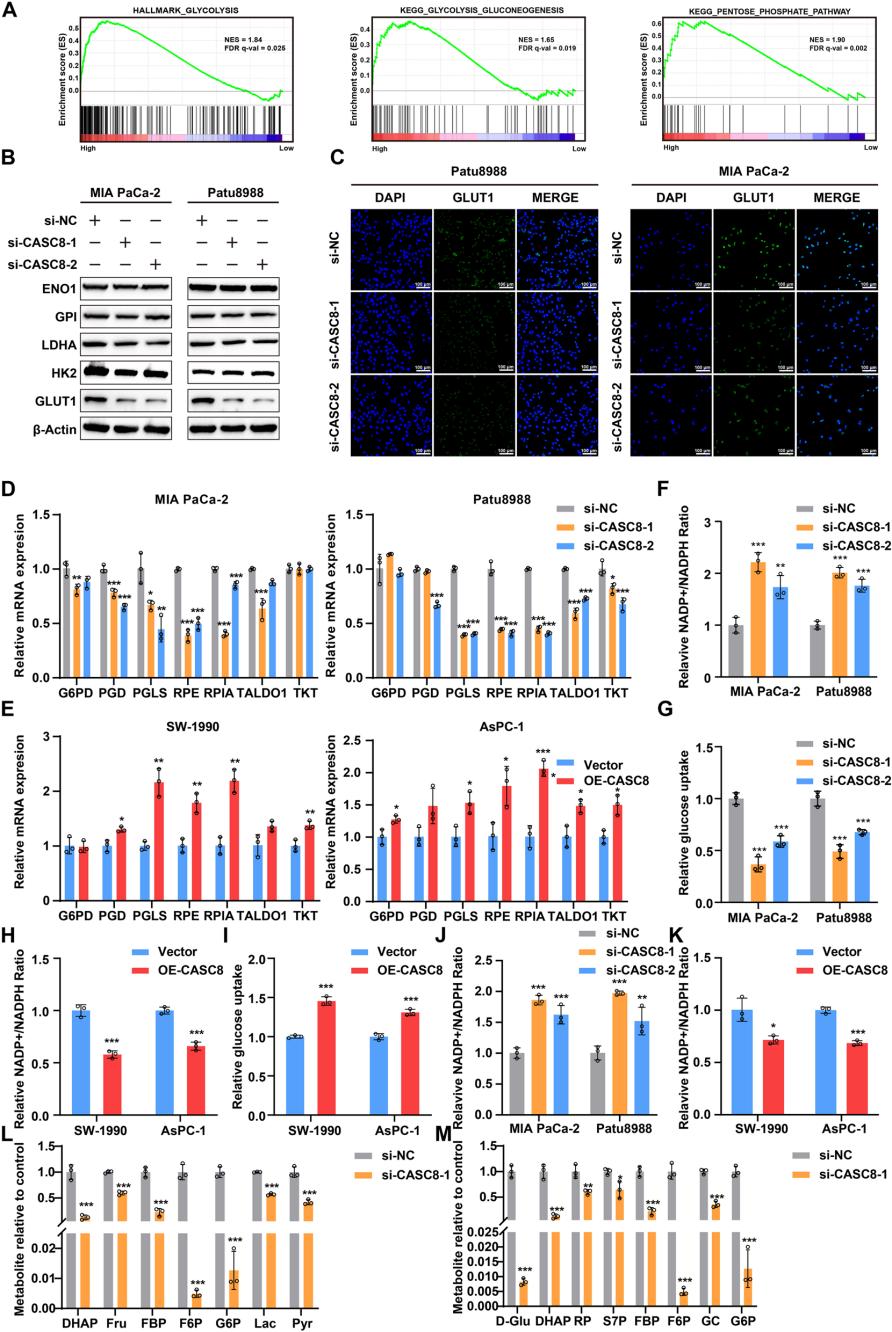
CASC8 is associated with the pentose phosphate pathway in PDAC cells. **A** GSEA analysis of groups expressing CASC8 at high and low levels in TCGA dataset by using hallmark gene sets and KEGG gene sets. **B** Western blotting analysis of glycolysis-related genes after CASC8 knockdown. **C** Immunofluorescence staining of GLUT1 in Patu8988 and MIA PaCa-2 after CASC8 knockdown. Scale bar = 100 μm. **D** qPCR analysis of genes involved in phosphate pentose pathway in MIA PaCa-2 and Patu8988 after CASC8 knockdown. Statistical significances were calculated by one-way ANOVA. **E** qPCR analysis of genes involved in phosphate pentose pathway in SW-1990 and AsPC-1 after CASC8 overexpression. Statistical significances were calculated by unpaired t-test. **F**, **G** Relative NADP^+^/NADPH ratio (**F**) and glucose uptake (**G**) in Patu8988 and MIA PaCa-2 after CASC8 knockdown. Statistical significances were calculated by one-way ANOVA. **H**, **I** Relative NADP.^+^/NADPH ratio (**H**) and glucose uptake (**I**) in SW-1990 and AsPC-1 after CASC8 overexpression. Statistical significances were calculated by unpaired t-test. **J** Relative NADP⁺/NADPH ratio in cells with CASC8 knockdown under glucose starvation conditions. Statistical significances were calculated by one-way ANOVA. **K** Relative NADP⁺/NADPH ratio in cells with CASC8 overexpression under glucose starvation conditions. Statistical significances were calculated by unpaired t-test. **L**, **M** The levels of glycolysis metabolites (**L**) and the intermediates of phosphate pentose pathway (**M**) in MIA PaCa-2 with CASC8 knockdown under glucose starvation conditions. All metabolite levels were normalized to the control cells. Statistical significances were calculated by unpaired t-test. DHAP, dihydroxyacetone phosphate; Fru, fructose; FBP, fructose 1,6-bisphosphate; F6P, fructose 6-phosphate; G6P, glucose 6-phosphate; Lac, lactate; Pyr, pyruvate; D-Glu, D-glucose; RP, D-ribulose 5-phosphate; S7P, D-sedoheptulose 7-phosphate; GC, gluconic acid. ***P < 0.001; **P < 0.01; *P < 0.05

To elucidate the underlying mechanisms by which CASC8 regulates PDAC progression and disulfidptosis, RNA sequencing (RNA-seq) based on three CASC8 knockdown cells and their control counterparts were performed (Supplementary Fig. 5A-B). The Gene Ontology (GO) and Kyoto Encyclopedia of Genes and Genomes (KEGG) enrichment analysis suggested that genes upregulated in negative control group were enriched in DNA replication, cell migration, and pentose phosphate pathway (Supplementary Fig. 5C-E). Besides, gene set enrichment analysis (GSEA) revealed that the glycolysis and pentose phosphate pathway were primarily enriched in the control group, which was supported by TCGA-PAAD data (Supplementary Fig. 5F, Fig. 5A). These findings led us to hypothesize that CASC8 may be involved in regulating glycolysis and pentose phosphate pathway in PDAC. To investigate whether CASC8 knockdown impacted the glycolysis pathway, we performed qRT-PCR analysis on five glycolysis-related genes. The results demonstrated that CASC8 knockdown significantly downregulated the expression of ENO1, GLUT1, and LDHA, regardless of the presence of glucose (Supplementary Fig. 6A-D). Conversely, CASC8 overexpression led to a significant upregulation of these same genes under both conditions (Supplementary Fig. 6E-H). Western blotting and cell immunofluorescence assays further corroborated these findings, revealing a decrease in GLUT1 protein expression following CASC8 knockdown (Fig. 5B-C). Collectively, these data suggest that CASC8 regulates the expression of GLUT1, potentially influencing downstream glucose metabolism processes.

The PPP is a critical metabolic pathway for glucose metabolism in all organisms. Within the PPP, glucose undergoes a series of enzymatic reactions to generate intermediates like phosphoenolpyruvate, ultimately leading to the production of pyruvate and glucose-6-phosphate. Notably, the PPP is the primary source of cytosolic NADPH [24]. Previous research has established that NADPH serves as a crucial reducing agent, helping to mitigate disulfide stress and promote cell survival. To investigate whether CASC8 inhibits PDAC cell disulfidptosis via the PPP, we analyzed the mRNA expression levels of seven key genes involved in this pathway. Our findings revealed that CASC8 depletion resulted in the downregulation of the majority of these genes, while CASC8 overexpression led to their upregulation (Fig. 5D-E). Similarly, the results remained consistent under glucose starvation conditions (Supplementary Fig. 6I-L). We further examined the relative NADP^+^/NADPH ratio and glucose uptake across the different groups. As expected, CASC8 knockdown increased the relative NADP^+^/NADPH ratio and decreased relative glucose uptake, while CASC8 overexpression exhibited the opposite effect (Fig. 5F-I). Additionally, we assessed the relative NADP^+^/NADPH ratio in cells exhibiting CASC8 knockdown and overexpression under glucose starvation conditions, and the results were consistent with those previously reported (Fig. 5J-K). To further explore the effects of CASC8 on cellular metabolism, we screened the metabolites by mass spectrometry in CASC8 knockdown MIA PaCa-2 cells and CASC8 overexpression SW-1990 cells under glucose starvation conditions. Metabolomics analysis revealed significant differences in metabolite levels between the control and CASC8 knockdown cells (Supplementary Fig. 7A-C). Both KEGG and MSEA enrichment analyses indicated that the upregulated metabolites in the control and CASC8 overexpression cells were enriched in glycolysis and the pentose phosphate pathway (Supplementary Fig. 7D-G). The levels of metabolites in glycolysis and the pentose phosphate pathway decreased following CASC knockdown and increased after CASC8 overexpression (Fig. 5L-M, Supplementary Fig. 7H-I). Collectively, these results suggest that CASC8 significantly impacts the pentose phosphate pathway in PDAC cells.

### CASC8 is capable of binding to c-Myc and modulating its protein stability

**Fig. 6:**
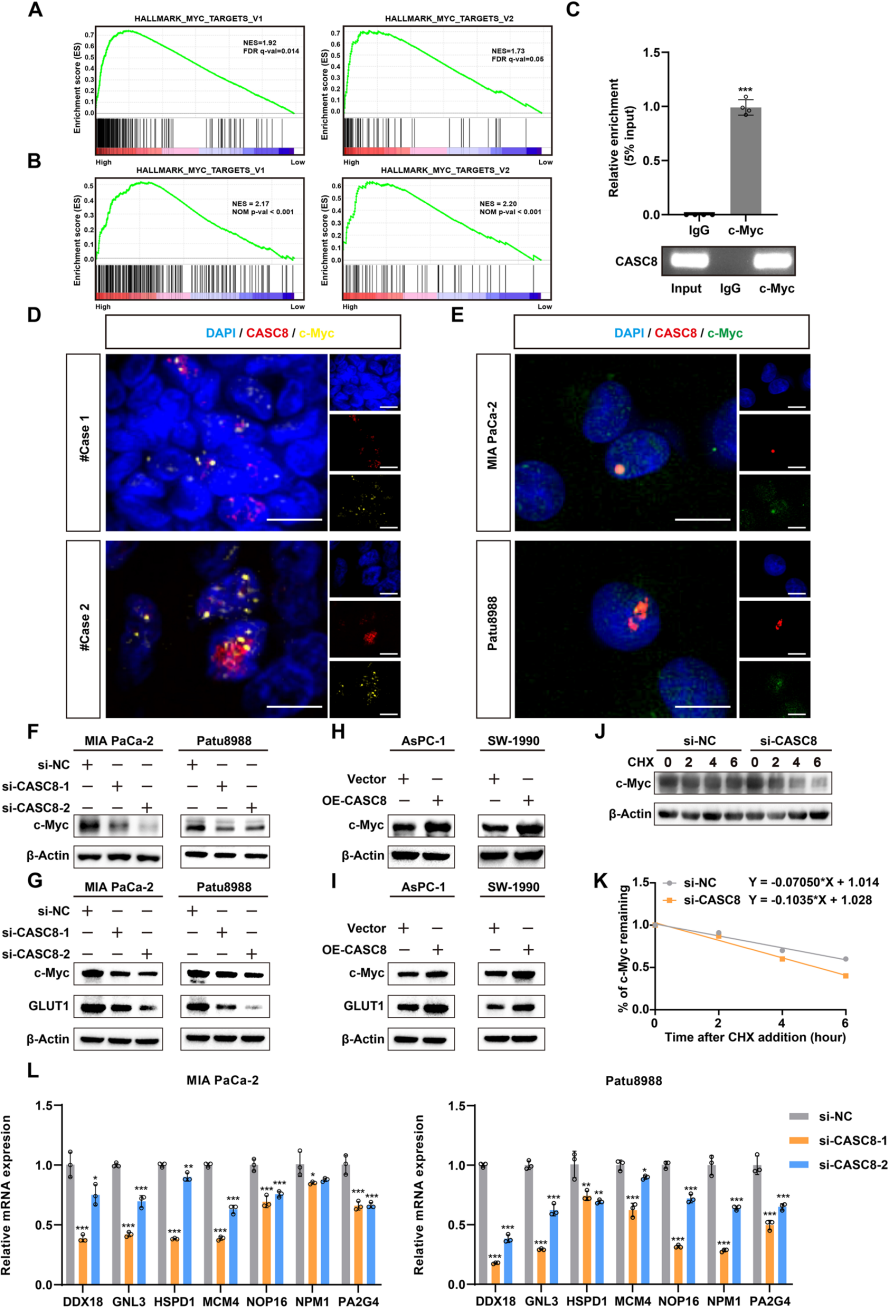
CASC8 is capable of binding to c-Myc and modulating its protein stability. **A** GSEA analysis of groups expressing CASC8 at high and low levels in TCGA dataset by using hallmark gene sets. **B** GSEA analysis between the control cells and CASC8 knockdown cells based on the results of RNA-seq by using hallmark gene sets. **C** RIP analysis of c-Myc binding to sequences of CASC8 with an anti-c-Myc antibody or IgG. Statistical significances were calculated by unpaired t-test. **D** Immunofluorescence staining of c-Myc and FISH of CASC8 in PDAC tissues. The results showed co-localization of c-Myc with CASC8 in the nucleus of cancer cells. Scale bar = 10 μm. **E** Immunofluorescence staining of c-Myc and FISH of CASC8 in MIA PaCa-2 and Patu8988. The results showed co-localization of c-Myc with CASC8 in the nucleus of PDAC cells. Scale bar = 20 μm. **F** Western blotting analysis of c-Myc after CASC8 knockdown in MIA PaCa-2 and Patu8988. **G** Western blotting analysis of c-Myc and GLUT1 after CASC8 knockdown in MIA PaCa-2 and Patu8988 under glucose starvation conditions. **H** Western blotting analysis of c-Myc after CASC8 overexpression in AsPC-1 and SW-1990. **I** Western blotting analysis of c-Myc and GLUT1 after CASC8 overexpression in AsPC-1 and SW-1990 under glucose starvation conditions. **J** Western blotting analysis of c-Myc after CASC8 knockdown and with 10 μg/mL CHX for six hours. **K** Determination of c-Myc stability in CASC8 knockdown and control cells incubated with 10 μg/mL CHX for six hours. **L** qPCR analysis of c-Myc target genes in MIA PaCa-2 and Patu8988 after CASC8 knockdown. Statistical significances were calculated by one-way ANOVA. ***P < 0.001; **P < 0.01; *P < 0.05

To elucidate the potential mechanisms by which increased CASC8 expression contributes to the pentose phosphate pathway, we performed GSEA on the TCGA-PAAD dataset. This analysis revealed a striking correlation between CASC8 expression and the expression of c-Myc target genes (Fig. 6A). This result was further confirmed by the RNA sequencing (Fig. 6B). The c-Myc transcription factor plays a pivotal role in regulating essential cellular processes. In tumor cells, rapid proliferation and growth often lead to an increased demand for energy. To meet this demand, tumors frequently exhibit overexpression or aberrant activation of c-Myc, which in turn modulates glucose metabolic pathways like glycolysis and the pentose phosphate pathway to provide ample energy to cancer cells. Based on these observations, we hypothesized that CASC8 might interact with the c-Myc protein, potentially activating downstream effector molecules to exert its function. RNA immunoprecipitation (RIP) and co-localization assays provided preliminary support for this hypothesis (Fig. 6C-E). Western blotting analysis further revealed a significant reduction in c-Myc protein levels following CASC8 knockdown, while CASC8 overexpression resulted in elevated c-Myc protein levels, regardless of the presence of glucose (Fig. 6F-I). To investigate protein stability, we treated cells with 10 μg/mL of cycloheximide (CHX) to inhibit protein synthesis for six hours. This experiment demonstrated a markedly increased degradation rate of c-Myc in CASC8 knockdown cells compared to controls (Fig. 6J-K). Additionally, knocking down CASC8 significantly decreased the mRNA levels of downstream c-Myc target genes (Fig. 6L). Collectively, these findings suggest that CASC8 can bind to the c-Myc protein, promoting its stability and thereby activating downstream effector molecules.

### CASC8 promotes PDAC growth and migration by regulating c-Myc in vitro

**Fig. 7:**
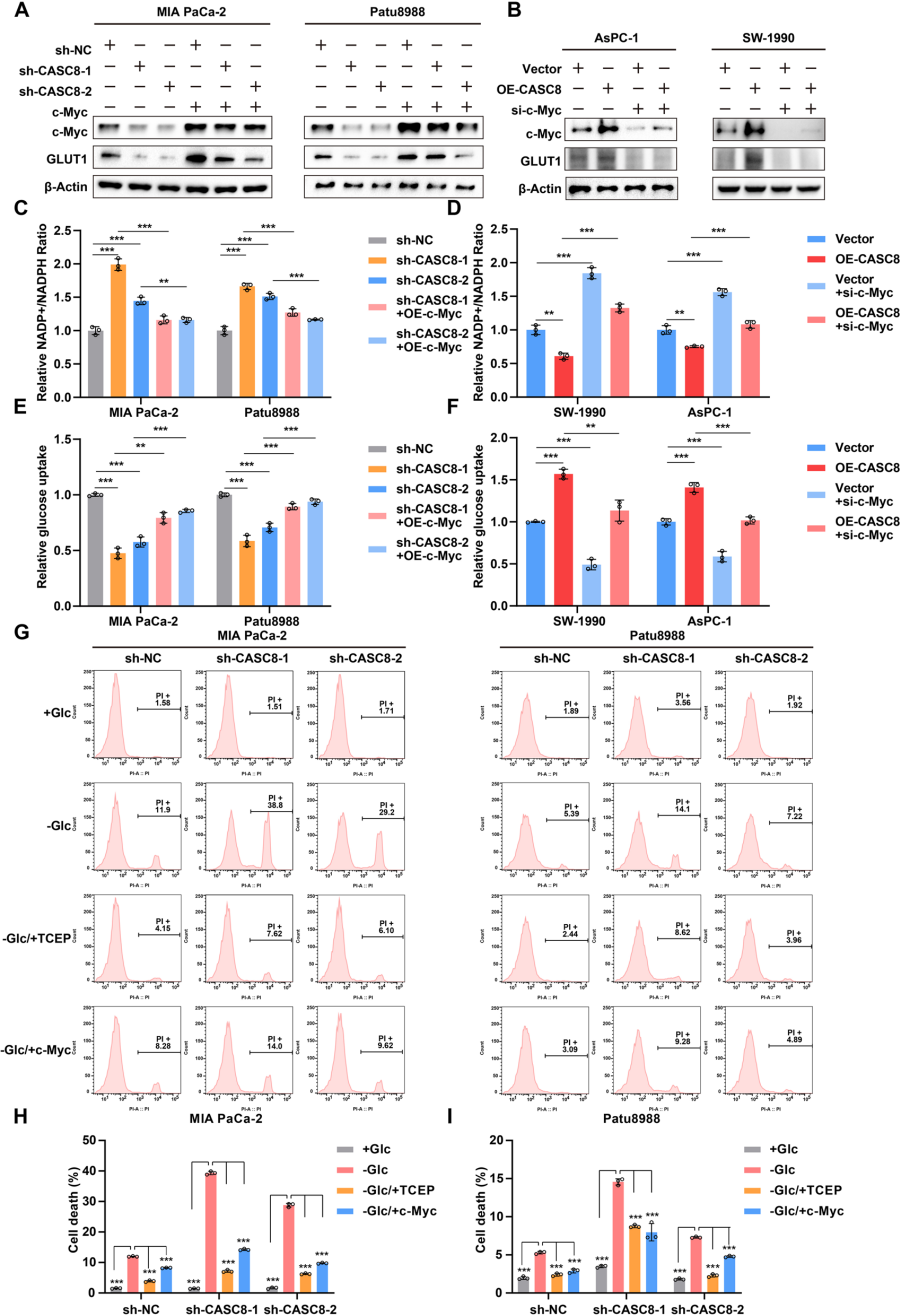
CASC8 inhibits PDAC cells disulfidptosis via c-Myc. **A** Western blotting analysis of c-Myc and GLUT1 following the c-Myc overexpression in CASC8 knockdown cells. **B** Western blotting analysis of c-Myc and GLUT1 following the c-Myc knockdown in CASC8 overexpression cells. **C** Relative NADP^+^/NADPH ratio in MIA PaCa-2 and Patu8988. CASC8 was knocked down in the cells, followed by the c-Myc overexpression. *P*-values between the two interested groups were calculated by unpaired t-test. **D** Relative NADP.^+^/NADPH ratio in SW-1990 and AsPC-1. CASC8 was overexpressed in the cells, followed by the c-Myc knockdown. *P*-values between the two interested groups were calculated by unpaired t-test. **E** Relative glucose uptake in MIA PaCa-2 and Patu8988. CASC8 was knocked down in the cells, followed by the c-Myc overexpression. *P*-values between the two interested groups were calculated by unpaired t-test. **F** Relative glucose uptake in SW-1990 and AsPC-1. CASC8 was overexpressed in the cells, followed by the c-Myc knockdown. *P*-values between the two interested groups were calculated by unpaired t-test. **G** The effect of c-Myc overexpression in CASC8 knockdown cells on cell death after cultured in glucose-free medium for 12 h and with or without 1 mM TCEP. **H**, I Statistical analysis of the PI-positive cells in MIA PaCa-2 (**H**) and Patu8988 (**I**) after CASC8 knockdown and following c-Myc overexpression. Statistical significances were calculated by one-way ANOVA. ***P < 0.001; **P < 0.01; *P < 0.05

To investigate the functional relationship between CASC8 and c-Myc in PDAC progression, we employed c-Myc overexpression in CASC8 knockdown cells and conversely knocked down c-Myc in CASC8 overexpression cells. Western blotting analysis confirmed the effectiveness of these manipulations (Fig. 7A-B). The colony formation assay revealed that c-Myc overexpression partially rescued the reduced proliferative capacity observed in CASC8 knockdown cells (Supplementary Fig. 8A-B). Conversely, c-Myc knockdown significantly diminished the enhanced proliferation observed in CASC8 overexpression cells (Supplementary Fig. 8C-D). Furthermore, the transwell assay demonstrated a link between CASC8 expression and c-Myc in PDAC cell migration. In CASC8 knockdown cells, c-Myc overexpression partially restored migratory capacity, while c-Myc knockdown in CASC8 overexpression cells resulted in decreased migration (Supplementary Fig. 8E-H). Collectively, these findings strongly suggest that CASC8 promotes PDAC progression in vitro through a c-Myc-dependent mechanism.

### CASC8 inhibits PDAC cell disulfidptosis via c-Myc in vitro

Our previous findings suggested that CASC8 regulates the pentose phosphate pathway in PDAC cells, potentially contributing to the inhibition of disulfidptosis. To further elucidate the role of c-Myc in this process, we investigated the relative NADP^+^/NADPH ratio and glucose uptake in different PDAC cell groups. We observed that c-Myc overexpression in CASC8 knockdown cells attenuated the increase in the NADP^+^/NADPH ratio and rescued the decrease in relative glucose uptake observed upon CASC8 depletion (Fig. 7C-D). Conversely, knocking down c-Myc in CASC8 overexpression cells resulted in the opposite effect (Fig. 7E-F). Next, we assessed the proportion of PI-positive cells in different treatment groups under glucose starvation conditions. The results demonstrated that c-Myc overexpression in CASC8 knockdown cells significantly rescued the increase in disulfidptosis cells observed upon CASC8 depletion (Fig. 7G-I). Furthermore, c-Myc knockdown in CASC8 overexpression cells increased disulfidptosis cells (Supplementary Fig. 9A-B). Collectively, these findings suggest that CASC8 promotes PDAC cell survival under glucose starvation conditions through a c-Myc-dependent mechanism.

### CASC8 inhibits PDAC disulfidptosis via c-Myc in vivo

**Fig. 8:**
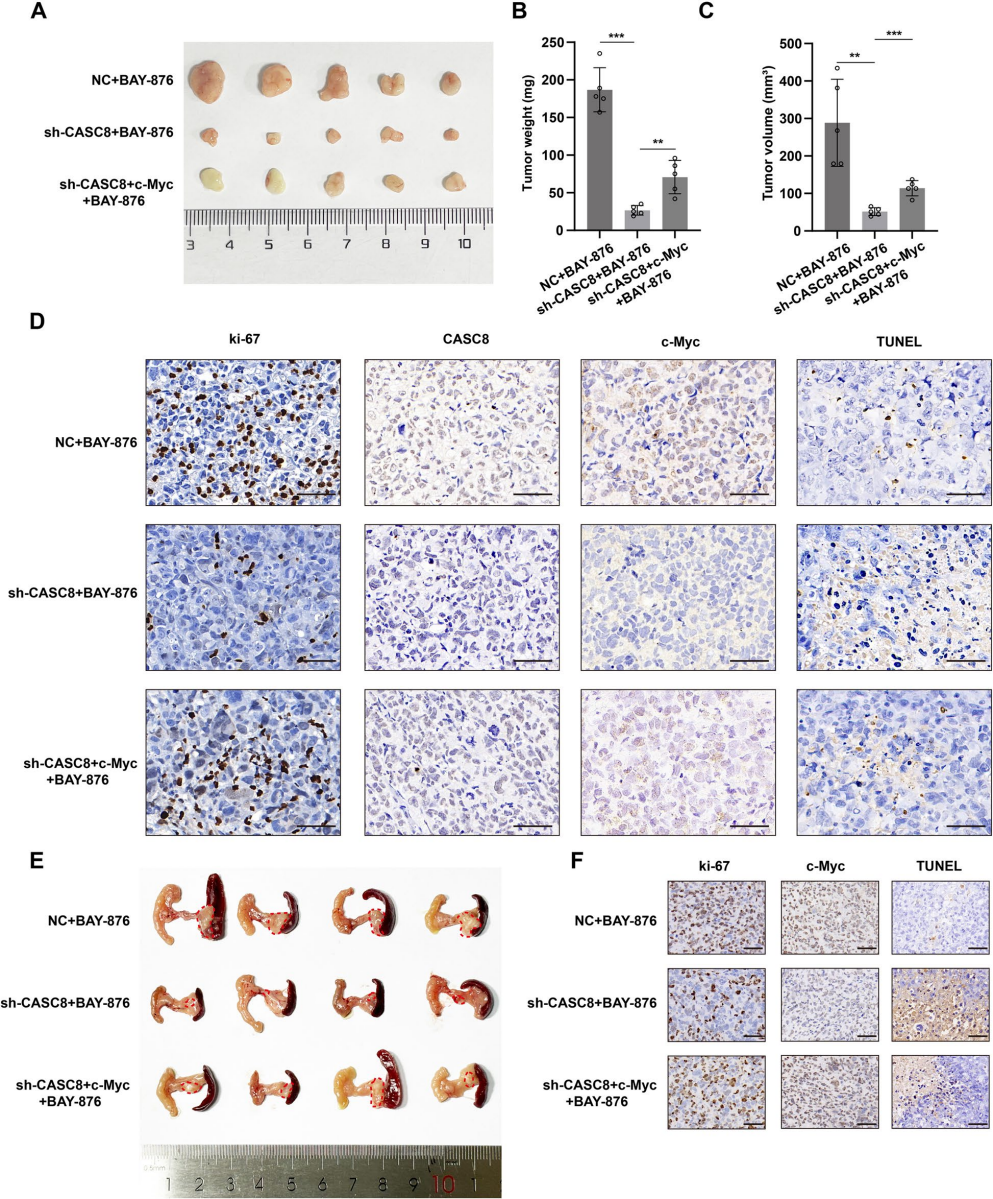
CASC8 inhibits PDAC disulfidptosis via c-Myc in vivo. **A** Subcutaneous xenograft tumor growth in mice inoculated with shNC, sh-CASC8, or sh-CASC8 + c-Myc MIA PaCa-2 (*n* = 5 for each group). After implanting the tumor subcutaneously for 7 days, all mice received intraperitoneal injections of BAY-876 at a dose of 3 mg/kg in 100 μL of 40% dimethylsulfoxide in saline (vehicle) or vehicle alone every two days. **B**, **C** Statistical analysis of tumor weight and volume of subcutaneous xenograft tumors from different groups. *P*-values between the two interested groups were calculated by unpaired t-test. **D** Representative images of IHC staining for ki-67, c-Myc, and CISH for CASC8, and TUNEL assay in subcutaneous xenograft tumors. Scale bar = 20 μm. **E** Orthotopic xenograft tumor growth in mice inoculated with shNC, sh-CASC8, or sh-CASC8 + c-Myc MIA PaCa-2 (*n* = 4 for each group). After implanting the tumor orthotopically for 7 days, all mice received intraperitoneal injections of BAY-876 at a dose of 3 mg/kg in 100 μL of 40% dimethylsulfoxide in saline (vehicle) or vehicle alone every two days. **F** Representative images of IHC staining for Ki-67, c-Myc, and TUNEL assay in orthotopic xenograft tumors. Scale bar = 20 μm. ***P < 0.001; **P < 0.01; *P < 0.05

Our previous in vitro studies suggested that CASC8 inhibits PDAC disulfidptosis through c-Myc. To further validate c-Myc as the critical downstream effector of CASC8 in PDAC disulfidptosis in vivo, we employed a subcutaneous xenograft model. Five mice inoculated with sh-CASC8 + c-Myc cells exhibited significantly increased tumor volume and weight compared to the sh-CASC8 group upon BAY-876 injection (Fig. 8A-C). Paraffin-embedded xenograft tumors were subjected to IHC staining for Ki-67, c-Myc, and CISH for CASC8, along with TUNEL assays. The sh-CASC8 + c-Myc group displayed a higher percentage of Ki-67-positive cells and fewer dead cells compared to the sh-CASC8 group (Fig. 8D). These findings were corroborated by the orthotopic xenograft model, where overexpression of c-Myc resulted in increased tumor burden in four mice harboring CASC8 knockdown cells (Fig. 8E-F). Collectively, these in vivo data strongly suggest that c-Myc functions as a key downstream effector of CASC8 in regulating disulfidptosis.

## Discussion

Regulated cell death (RCD) plays a critical role not only in organismal development and homeostasis but also in tumorigenesis [25, 26]. RCD functions as a tumor suppressive mechanism by initiating programmed cell death in cancer cells, thereby preventing their uncontrolled proliferation [27, 28]. In PDAC, a hallmark feature of the tumor microenvironment is the combination of poor vascularization and excessive desmoplasia, which ultimately subject cancer cells to severe metabolic stress [29, 30]. This accumulated metabolic stress can trigger RCD pathways, leading to the elimination of cancer cells [31]. Consequently, targeting RCD with small-molecule compounds has emerged as a promising therapeutic strategy for various cancers [32]. Ferroptosis, a form of RCD, is activated by the accumulation of lipid-derived reactive oxygen species (ROS) [33]. Studies have shown that targeting the cysteine import pathway to induce ferroptosis can inhibit tumor growth in pancreatic cancer [34]. Necroptosis, another form of RCD, also holds promise as a therapeutic target for eliminating cancer cells and preventing tumorigenesis [35]. Intra-tumoral activation of RIPK3, a key component of the necroptotic pathway, has been demonstrated to enhance anti-tumor immunity and promote tumor regression [36]. Taken together, these various RCD modalities are actively investigated due to their potential to improve cancer treatment efficacy [37].

Disulfidptosis, a recently discovered form of RCD, is linked to metabolic stress and the depletion of cellular reducing power. Studies have shown that high expression of SLC7A11 in kidney cancer cells leads to NADPH depletion under glucose starvation, ultimately triggering disulfidptosis-mediated cell death [38]. However, the prevalence and regulatory mechanisms of disulfidptosis in PDAC remain largely unexplored. In this study, we provide the first evidence for the involvement of disulfidptosis in PDAC cells. Our findings demonstrated that glucose starvation could significantly increase the proportion of dead cells, an effect reversed by the reducing agents DTT and TCEP, which protected cells from disulfide stress. Furthermore, we identified disulfidptosis-related lncRNAs and revealed the upregulation of CASC8 in PDAC. We propose that CASC8 promotes PDAC progression by activating the pentose phosphate pathway in a c-Myc-dependent manner, ultimately leading to the inhibition of disulfidptosis. These findings offer novel insights into the pathogenesis of PDAC and suggest the potential of targeting RCD pathways as a therapeutic strategy for this devastating disease.

Previous studies have reported that CASC8 is upregulated and promotes progression in various cancers [22, 39]. Our findings align with these observations, demonstrating that CASC8 functions as a disulfidptosis-related lncRNA and exerts an oncogenic role in PDAC under metabolic stress conditions. Notably, knocking down CASC8 expression significantly induced disulfidptosis in PDAC cells, an effect reversed by the reducing agents DTT and TCEP. Conversely, CASC8 overexpression yielded opposing results. These protective roles of CASC8 appear to be dependent on the pentose phosphate pathway, as revealed by both RNA sequencing and metabolic analyses. While not directly generating ATP, the pentose phosphate pathway plays a critical role in maintaining cellular carbon balance, supplying precursors for nucleotide and amino acid biosynthesis, providing reducing equivalents for synthetic processes, and combating oxidative stress [40]. Consequently, upregulation of the pentose phosphate pathway can protect cells from disulfide stress and ensure their survival under glucose starvation conditions [41]. These observations support the hypothesis that an enhanced pentose phosphate pathway promotes tumor cell survival in nutrient-depleted environments.

The c-Myc transcription factor plays a central role in regulating various cellular processes. Dysregulation of c-Myc, typically characterized by overexpression or aberrant activation, can induce uncontrolled cell proliferation, suppress apoptosis, and drive metabolic reprogramming. Consequently, c-Myc dysregulation is strongly linked to the pathogenesis of several diseases, particularly cancer [42, 43]. Notably, c-Myc activation is known to induce the expression of key enzymes involved in both glycolysis and the pentose phosphate pathway [44–46]. Our study identified c-Myc as a downstream target gene of CASC8, and genetic suppression of c-Myc exhibited tumor-suppressive effects in PDAC cells overexpressing CASC8. Furthermore, we demonstrated that CASC8-mediated metabolic alterations are dependent on c-Myc activity. Overexpression of c-Myc in CASC8 knockdown cells mitigated the increase in the NADP^+^/NADPH ratio and rescued the decrease in relative glucose uptake. These metabolic changes ultimately alleviated disulfide stress in PDAC cells and promoted their survival under glucose starvation conditions. However, the precise mechanisms underlying the interaction between CASC8 and c-Myc remain to be fully elucidated. Our findings suggest a potential direct interaction between CASC8 and c-Myc, potentially influencing the stability of the c-Myc protein. In support of this hypothesis, we identified an interaction complex between CASC8 and c-Myc. Nevertheless, the specific mechanisms of how CASC8 interacts with c-Myc and the location of the binding sites remain unclear. Further investigation is warranted to unravel this intricate regulatory complex.

## Conclusion

**Fig. 9:**
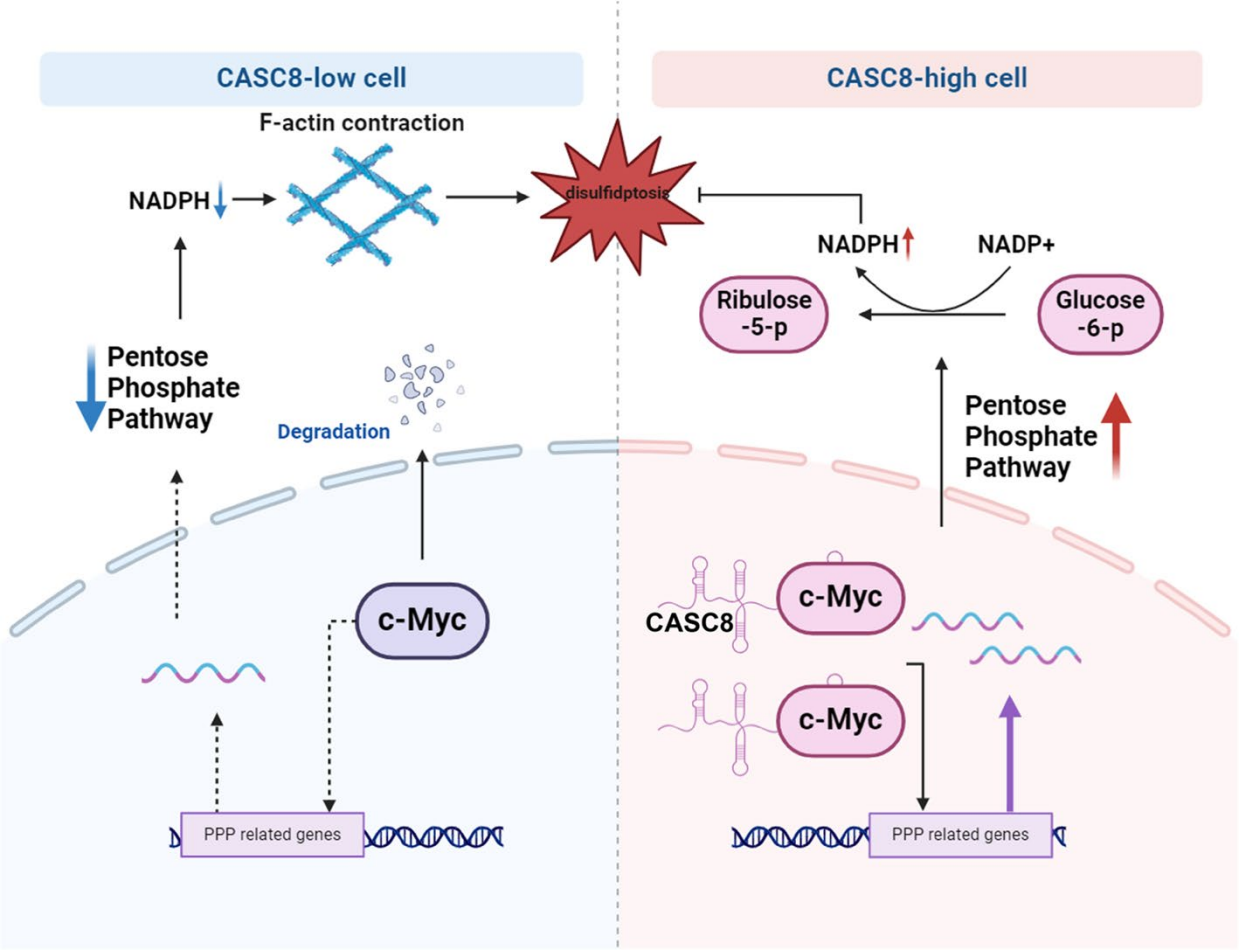
The mechanism diagram of this study. PDAC cells expressing CASC8 activate the pentose phosphate pathway to inhibit disulfidptosis in PDAC though its interaction with the c-Myc

In conclusion, our study identifies CASC8 as a critical regulator of PDAC cell survival through its interaction with the c-Myc transcription factor. Under metabolic stress conditions, PDAC cells expressing CASC8 exhibit an enhanced capacity for survival. This survival advantage is conferred by the upregulation of the pentose phosphate pathway, leading to increased NADPH production (Fig. 9). These findings highlight the pivotal role of CASC8 in regulating disulfidptosis and suggest CASC8 as a potential target for therapeutic intervention in PDAC.

## Supplementary Information

Supplementary Material 1: Supplementary Fig. 1. Construction of the disulfidptosis-related lncRNA signature in the training group. (A) Forest plot of DRLs from the results of univariate Cox regression analysis in the training group. (B) LASSO regression of the DRLs in the training group. (D) Expression level of five lncRNAs in each sample within the training group. (E–F) Distribution of the risk score and survival status in the high- and low-risk groups within the training group. (G-H) Kaplan–Meier survival curve analysis of OS (G) and DFS (H) between the high- and low-risk groups within the training group. (I) ROC curves for predicting the survival rates of patients in the training groups. (J-K) Univariate Cox analysis (J) and Multivariate Cox analysis (K) of clinicopathological features and risk score in the training group.

Supplementary Material 2: Supplementary Fig. 2. Validation of the disulfidptosis-related lncRNA signature in the testing group and entire cohort. (A-B) Expression level of five lncRNAs in the testing group (A) and entire cohort (B). (C-D) Distribution of the risk score in the testing group (C) and entire cohort (D). (E–F) Distribution of survival status in the testing group (E) and entire cohort (F). (G-J) Kaplan–Meier survival curve analysis of OS and DFS between the high- and low-risk groups in the testing group (G-H) and entire cohort (I-J). (K-L) ROC curves for predicting the survival rates of patients in the testing group (K) and entire cohort (L). (M-P) Univariate and multivariate Cox analysis of clinicopathological features and risk score in the testing group (M–N) and entire cohort (O-P).

Supplementary Material 3: Supplementary Fig. 3. The expression levels and prognostic values of four lncRNAs in PDAC. (A-D) The expression levels of four lncRNAs in TCGA-PAAD and GTEx-Pancreas datasets. P -values between the two groups were calculated by unpaired t-test. (E-L) Kaplan–Meier survival curve analysis of OS (E–H) and DFS (I-L) conducted between the high and low expression groups using data from TCGA.

Supplementary Material 4: Supplementary Fig. 4. CASC8 promotes PDAC growth and migration in vitro. (A) qPCR analysis of CASC8 in PDAC cell lines. (B) Knockdown efficiencies of CASC8 in MIA PaCa-2 and Patu8988 calculated by qPCR analysis. Statistical significances were calculated by one-way ANOVA. (C) Overexpression efficiencies of CASC8 in SW-1990 and AsPC-1 calculated by qPCR analysis. Statistical significances were calculated by unpaired t-test. (D) Relative cell viability of MIA PaCa-2 and Patu8988 after CASC8 knockdown. Statistical significances were calculated by two-way ANOVA. (E–F) Colony-formation assay (E) and statistical analysis (F) of MIA PaCa-2 and Patu8988 after CASC8 knockdown. Statistical significances were calculated by one-way ANOVA. (G) Relative cell viability of SW-1990 and AsPC-1 after CASC8 overexpression. Statistical significances were calculated by two-way ANOVA. (H-I) Colony-formation assay (H) and statistical analysis (I) of SW-1990 and AsPC-1 after CASC8 overexpression. Statistical significances were calculated by unpaired t-test. (J) Representative images of EdU assay conducted in MIA PaCa-2 and Patu8988 after CASC8 knockdown. Scale bar = 100 μm. (K-L) Flow cytometry assay (K) and statistical analysis (L) of EdU positive cells in SW-1990 and AsPC-1 after CASC8 overexpression. Statistical significances were calculated by unpaired t-test. (M–N) Cell migration assay (M) and statistical analysis (N) of MIA PaCa-2 and Patu8988 after CASC8 knockdown. Scale bar = 100 μm. Statistical significances were calculated by one-way ANOVA. (O-P) Cell migration assay (O) and statistical analysis (P) of SW-1990 and AsPC-1 after CASC8 overexpression. Scale bar = 100 μm. Statistical significances were calculated by unpaired t-test. *** *P* < 0.001; ** *P* < 0.01; * *P* < 0.05.

Supplementary Material 5: Supplementary Fig. 5. The enrichment analysis of the DEGs between the control cells and CASC8 knockdown cells. (A) Gene expression heatmap of the control cells and CASC8 knockdown cells. (B) Volcano plotting of the control cells and CASC8 knockdown cells. The most significantly upregulated or downregulated genes and CASC8 were marked. (C) The results of GO enrichment analysis in the control group compared to CASC8 knockdown group. (D) The results of KEGG enrichment analysis in the control group compared to CASC8 knockdown group. (E) Gene expression heatmap of genes involved in phosphate pentose pathway between the control cells and CASC8 knockdown cells. (F) GSEA analysis between the control cells and CASC8 knockdown cells by using hallmark gene sets and KEGG gene sets.

Supplementary Material 6: Supplementary Fig. 6. CASC8 knockdown or overexpression modulates the expression of genes involved in glycolysis and pentose phosphate pathway. (A-B) qPCR analysis of glycolysis-related genes in MIA PaCa-2 (A) and Patu8988 (B) after CASC8 knockdown. Statistical significances were calculated by one-way ANOVA. (C-D) qPCR analysis of glycolysis-related genes in MIA PaCa-2 (C) and Patu8988 (D) following CASC8 knockdown under conditions of glucose starvation. Statistical significances were calculated by one-way ANOVA. (E–F) qPCR analysis of glycolysis-related genes in SW-1990 (E) and AsPC-1 (F) after CASC8 overexpression. Statistical significances were calculated by unpaired t-test. (G-H) qPCR analysis of glycolysis-related genes in SW-1990 (G) and AsPC-1 (H) following CASC8 overexpression under conditions of glucose starvation. Statistical significances were calculated by unpaired t-test. (I-J) qPCR analysis of genes involved in phosphate pentose pathway in MIA PaCa-2 (I) and Patu8988 (J) following CASC8 knockdown under conditions of glucose starvation. Statistical significances were calculated by one-way ANOVA. (K-L) qPCR analysis of genes involved in phosphate pentose pathway in SW-1990 (K) and AsPC-1 (L) following CASC8 overexpression under conditions of glucose starvation. Statistical significances were calculated by unpaired t-test. *** *P* < 0.001; ** *P* < 0.01; * *P* < 0.05.

Supplementary Material 7: Supplementary Fig. 7. Metabolomics analysis identified the effects of CASC8 on cellular metabolism. (A) Metabolites levels heatmap of the control and CASC8 knockdown MIA PaCa-2 cells under glucose starvation conditions. (B) Results of the principal component analysis (PCA) for the control cells and CASC8 knockdown cells. (C) Volcano plot of differential metabolites between control cells and CASC8 knockdown cells. The most significantly upregulated or downregulated metabolites were marked. (D) The KEGG enrichment analysis of differential metabolites upregulated in the control cells. (E) The MSEA enrichment analysis of differential metabolites upregulated in the control cells. (F) The KEGG enrichment analysis of differential metabolites upregulated in the CASC8 overexpression cells. (G) The MSEA enrichment analysis of differential metabolites upregulated in the CASC8 overexpression cells. (H-I) The levels of glycolysis metabolites (H) and the intermediates of phosphate pentose pathway (I) in SW-1990 with CASC8 overexpression under glucose starvation conditions. All metabolite levels were normalized to the vector cells. Statistical significances were calculated by unpaired t-test. DHAP, dihydroxyacetone phosphate; Fru, fructose; FBP, fructose 1,6-bisphosphate; F6P, fructose 6-phosphate; G6P, glucose 6-phosphate; Lac, lactate; Pyr, pyruvate; 6PG, 6-phosphogluconate; RP, D-ribulose 5-phosphate; S7P, D-sedoheptulose 7-phosphate; GC, gluconic acid; RB, ribose; RBP, ribose 1,5-bisphosphate. *** *P* < 0.001; ** *P* < 0.01; * *P* < 0.05.

Supplementary Material 8: Supplementary Fig. 8. CASC8 promotes PDAC growth and migration by regulating c-Myc. (A-B) Colony-formation assay (A) and statistical analysis (B) of CASC8 knockdown cells after c-Myc overexpression. (C-D) Colony-formation assay (C) and statistical analysis (D) of CASC8 overexpression cells after c-Myc knockdown. (E–F) Cell migration assay (E) and statistical analysis (F) of CASC8 knockdown cells after c-Myc overexpression. (G-H) Cell migration assay (G) and statistical analysis (H) of CASC8 overexpression cells after c-Myc knockdown. Scale bar = 100 μm. Statistical significances were calculated by unpaired t-test. *** *P* < 0.001; ** *P* < 0.01; * *P* < 0.05.

Supplementary Material 9: Supplementary Fig. 9. CASC8 inhibits PDAC cells disulfidptosis via c-Myc. (A) The effects of c-Myc knockdown in CASC8 overexpression cells on cell death after cultured in glucose-free medium for 12 h and with or without 1 mM TCEP. (B) Statistical analysis of the PI-positive cells in SW-1990 and AsPC-1 after CASC8 overexpression and following c-Myc knockdown. P -values between the two interested groups were calculated by unpaired t-test. *** *P* < 0.001; ** *P* < 0.01; * *P* < 0.05.

Supplementary Material 10: Supplementary Table. The primers used in this study.

## Data Availability

The data are available within the Article, Supplementary Information, or available from the authors upon request. Source data are provided with this paper.

## Abbreviations

PDAC: Pancreatic ductal adenocarcinoma
CASC8: Cancer Susceptibility 8
TCGA: The Cancer Genome Atlas
NADPH: Nicotinamide adenine dinucleotide phosphate
PCD: Programmed cell death
PPP: Pentose phosphate pathway
GTEx: Genotype-Tissue Expression
DRG: Disulfidptosis-related gene
DRL: Disulfidptosis-related lncRNA
LASSO: Least absolute shrinkage and selection operator
ROC: Receiver operating characteristic
AUC: Area under the ROC curve
DMEM: Dulbecco's modified Eagle's medium
FBS: Fetal bovine serum
P/S: Penicillin/streptomycin
RPMI: Roswell Park Memorial Institute
qRT-PCR: Quantitative real-time PCR
CCK-8: Cell counting kit-8
SDS-PAGE: Sodium dodecyl sulfate–polyacrylamide gel electrophoresis
TBST: Tris-buffered saline with 0.1% Tween-20
BSA: Bovine serum albumin
SLC7A11: Solute Carrier Family 7 Member 11
GLUT1: Glucose Transporter Type 1
ENO1: Enolase 1
GPI: Glucose-6-Phosphate Isomerase
LDHA: Lactate Dehydrogenase A
HK2: Hexokinase 2
HRP: Horseradish peroxidase
ECL: Enhanced chemiluminescence
DTT: Dithiothreitol
TCEP: Tris-(2-carboxyethyl)-phosphine
PI: Propidium iodide
PBS: Phosphate-buffered saline
DAPI: 4',6-Diamidino-2-phenylindole
BCA: Bicinchoninic acid
IF: Immunofluorescence
CK19: Cytokeratin 19
IHC: Immunohistochemistry
TUNEL: Terminal deoxynucleotidyl transferase dUTP nick-end labeling
CISH: Chromogenic in situ hybridization
ANOVA: Analysis of Variance
CNV: Copy number variation
OS: Overall survival
PFS: Progression-free survival
DFS: Disease-free survival
FISH: Fluorescence in situ hybridization
siRNA: Short interfering RNA
shRNA: Short hairpin RNA
RNA-seq: RNA sequencing
GO: Gene Ontology
KEGG: Kyoto Encyclopedia of Genes and Genomes
GSEA: Gene set enrichment analysis
RIP: RNA immunoprecipitation
CHX: Cycloheximide
RCD: Regulated cell death
ROS: Reactive oxygen species
