# Supplementary figures and images for: CASC8 activates the pentose phosphate pathway to inhibit disulfidptosis in pancreatic ductal adenocarcinoma though the c-Myc-GLUT1 axis
Source: J Exp Clin Cancer Res. 2025 Jan 27;44:26. doi: 10.1186/s13046-025-03295-w (PMC11771065; doi:10.1186/s13046-025-03295-w)

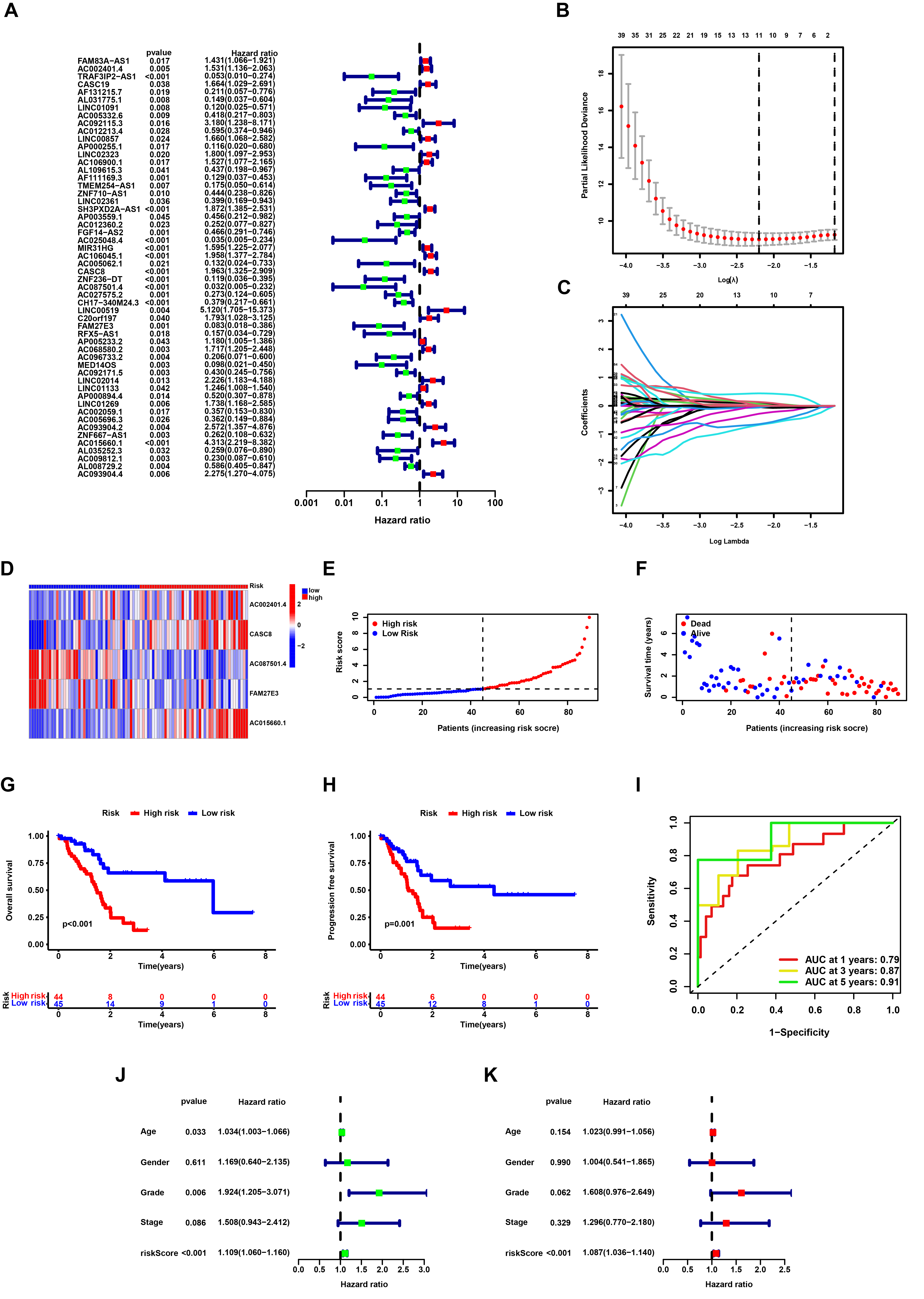

Supplement: Supplementary file 1 — Supplementary Material 1: Supplementary Fig. 1. Construction of the disulfidptosis-related lncRNA signature in the training group. (A) Forest plot of DRLs from the results of univariate Cox regression analysis in the training group. (B) LASSO regression of the DRLs in the training group. (D) Expression level of five lncRNAs in each sample within the training group. (E–F) Distribution of the risk score and survival status in the high- and low-risk groups within the training group. (G-H) Kaplan–Meier survival curve analysis of OS (G) and DFS (H) between the high- and low-risk groups within the training group. (I) ROC curves for predicting the survival rates of patients in the training groups. (J-K) Univariate Cox analysis (J) and Multivariate Cox analysis (K) of clinicopathological features and risk score in the training group. [file 13046_2025_3295_MOESM1_ESM.jpg]

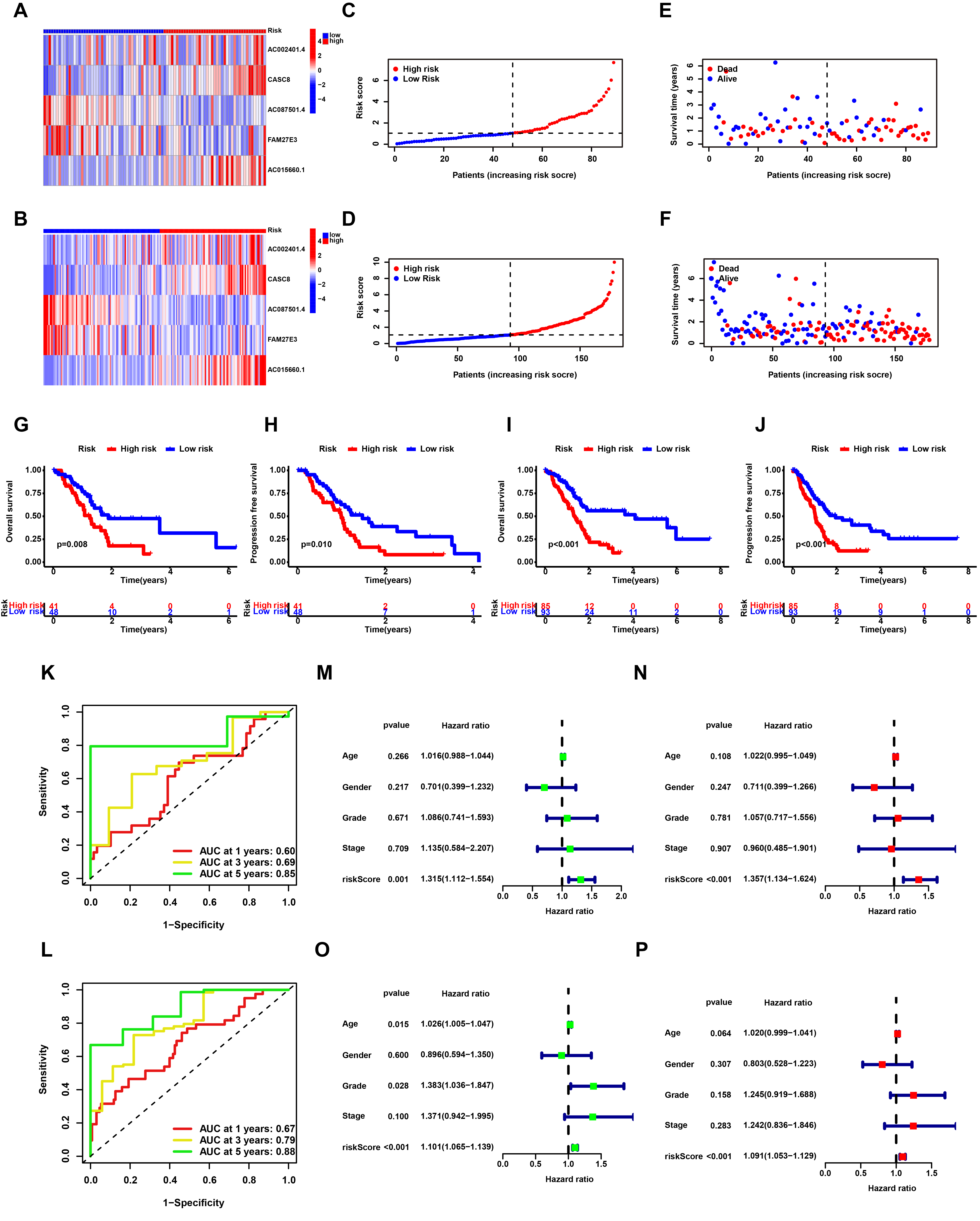

Supplement: Supplementary file 2 — Supplementary Material 2: Supplementary Fig. 2. Validation of the disulfidptosis-related lncRNA signature in the testing group and entire cohort. (A-B) Expression level of five lncRNAs in the testing group (A) and entire cohort (B). (C-D) Distribution of the risk score in the testing group (C) and entire cohort (D). (E–F) Distribution of survival status in the testing group (E) and entire cohort (F). (G-J) Kaplan–Meier survival curve analysis of OS and DFS between the high- and low-risk groups in the testing group (G-H) and entire cohort (I-J). (K-L) ROC curves for predicting the survival rates of patients in the testing group (K) and entire cohort (L). (M-P) Univariate and multivariate Cox analysis of clinicopathological features and risk score in the testing group (M–N) and entire cohort (O-P). [file 13046_2025_3295_MOESM2_ESM.jpg]

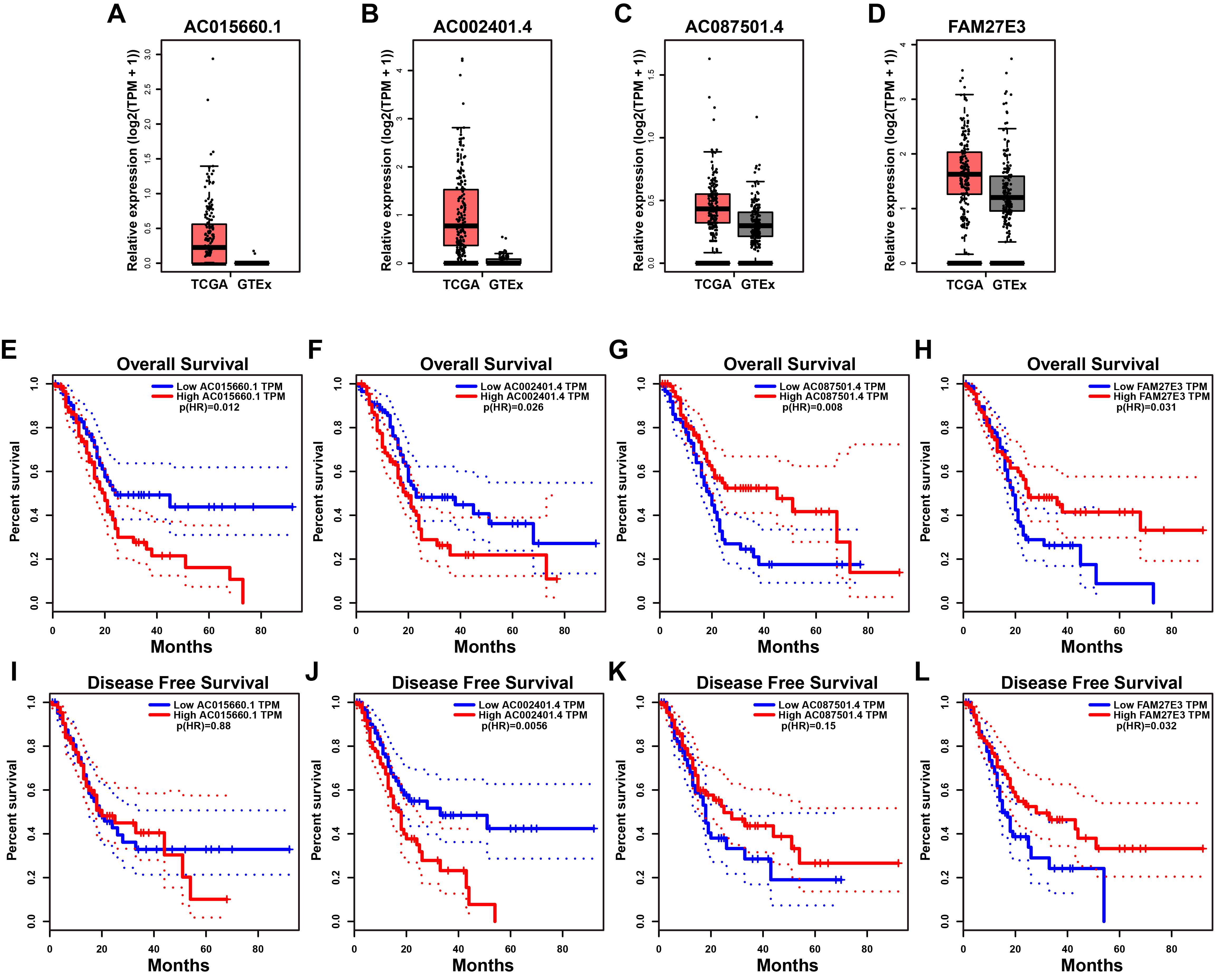

Supplement: Supplementary file 3 — Supplementary Material 3: Supplementary Fig. 3. The expression levels and prognostic values of four lncRNAs in PDAC. (A-D) The expression levels of four lncRNAs in TCGA-PAAD and GTEx-Pancreas datasets. P -values between the two groups were calculated by unpaired t-test. (E-L) Kaplan–Meier survival curve analysis of OS (E–H) and DFS (I-L) conducted between the high and low expression groups using data from TCGA. [file 13046_2025_3295_MOESM3_ESM.jpg]

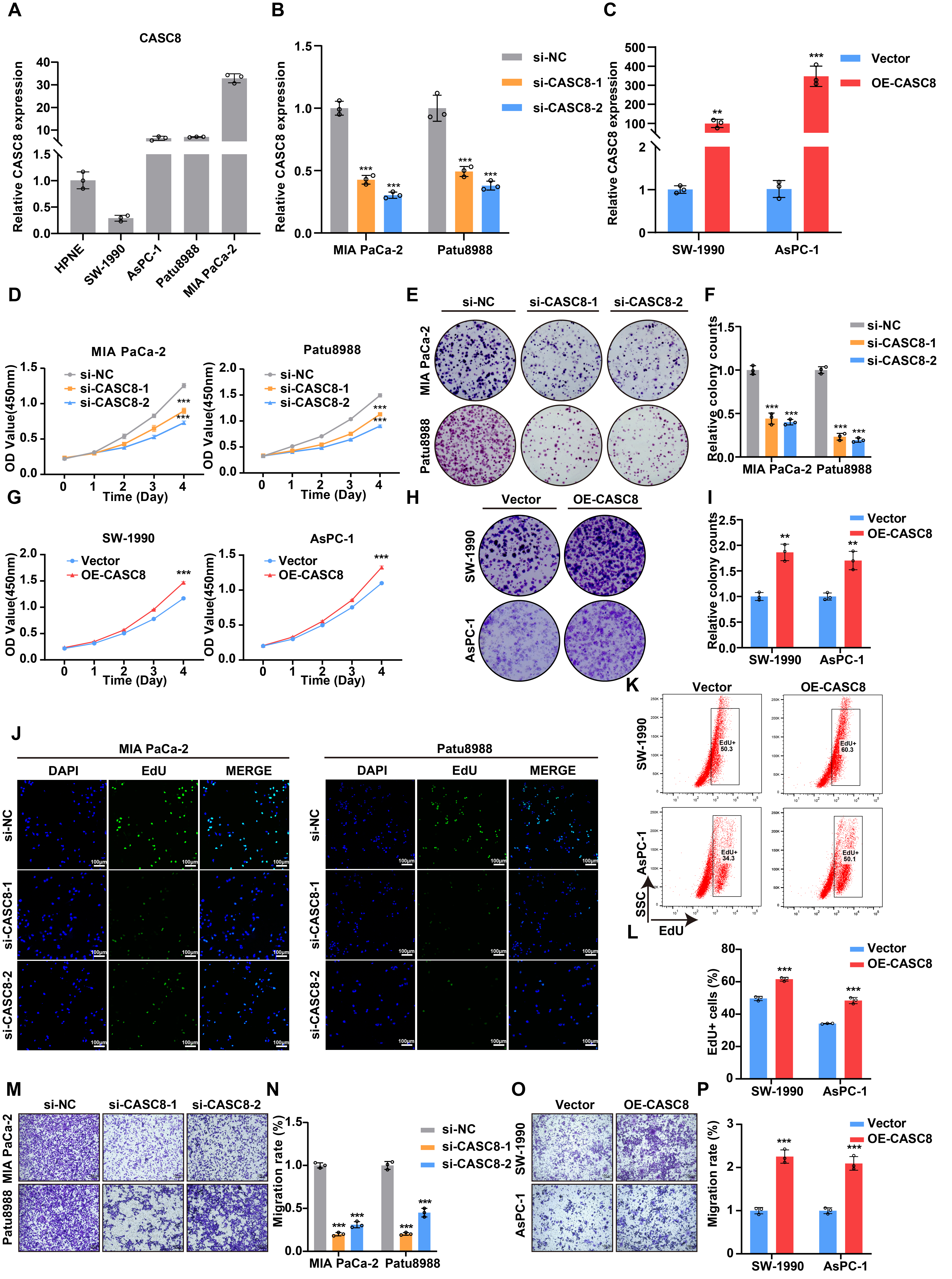

Supplement: Supplementary file 4 — Supplementary Material 4: Supplementary Fig. 4. CASC8 promotes PDAC growth and migration in vitro. (A) qPCR analysis of CASC8 in PDAC cell lines. (B) Knockdown efficiencies of CASC8 in MIA PaCa-2 and Patu8988 calculated by qPCR analysis. Statistical significances were calculated by one-way ANOVA. (C) Overexpression efficiencies of CASC8 in SW-1990 and AsPC-1 calculated by qPCR analysis. Statistical significances were calculated by unpaired t-test. (D) Relative cell viability of MIA PaCa-2 and Patu8988 after CASC8 knockdown. Statistical significances were calculated by two-way ANOVA. (E–F) Colony-formation assay (E) and statistical analysis (F) of MIA PaCa-2 and Patu8988 after CASC8 knockdown. Statistical significances were calculated by one-way ANOVA. (G) Relative cell viability of SW-1990 and AsPC-1 after CASC8 overexpression. Statistical significances were calculated by two-way ANOVA. (H-I) Colony-formation assay (H) and statistical analysis (I) of SW-1990 and AsPC-1 after CASC8 overexpression. Statistical significances were calculated by unpaired t-test. (J) Representative images of EdU assay conducted in MIA PaCa-2 and Patu8988 after CASC8 knockdown. Scale bar = 100 μm. (K-L) Flow cytometry assay (K) and statistical analysis (L) of EdU positive cells in SW-1990 and AsPC-1 after CASC8 overexpression. Statistical significances were calculated by unpaired t-test. (M–N) Cell migration assay (M) and statistical analysis (N) of MIA PaCa-2 and Patu8988 after CASC8 knockdown. Scale bar = 100 μm. Statistical significances were calculated by one-way ANOVA. (O-P) Cell migration assay (O) and statistical analysis (P) of SW-1990 and AsPC-1 after CASC8 overexpression. Scale bar = 100 μm. Statistical significances were calculated by unpaired t-test. *** P < 0.001; ** P < 0.01; * P < 0.05. [file 13046_2025_3295_MOESM4_ESM.jpg]

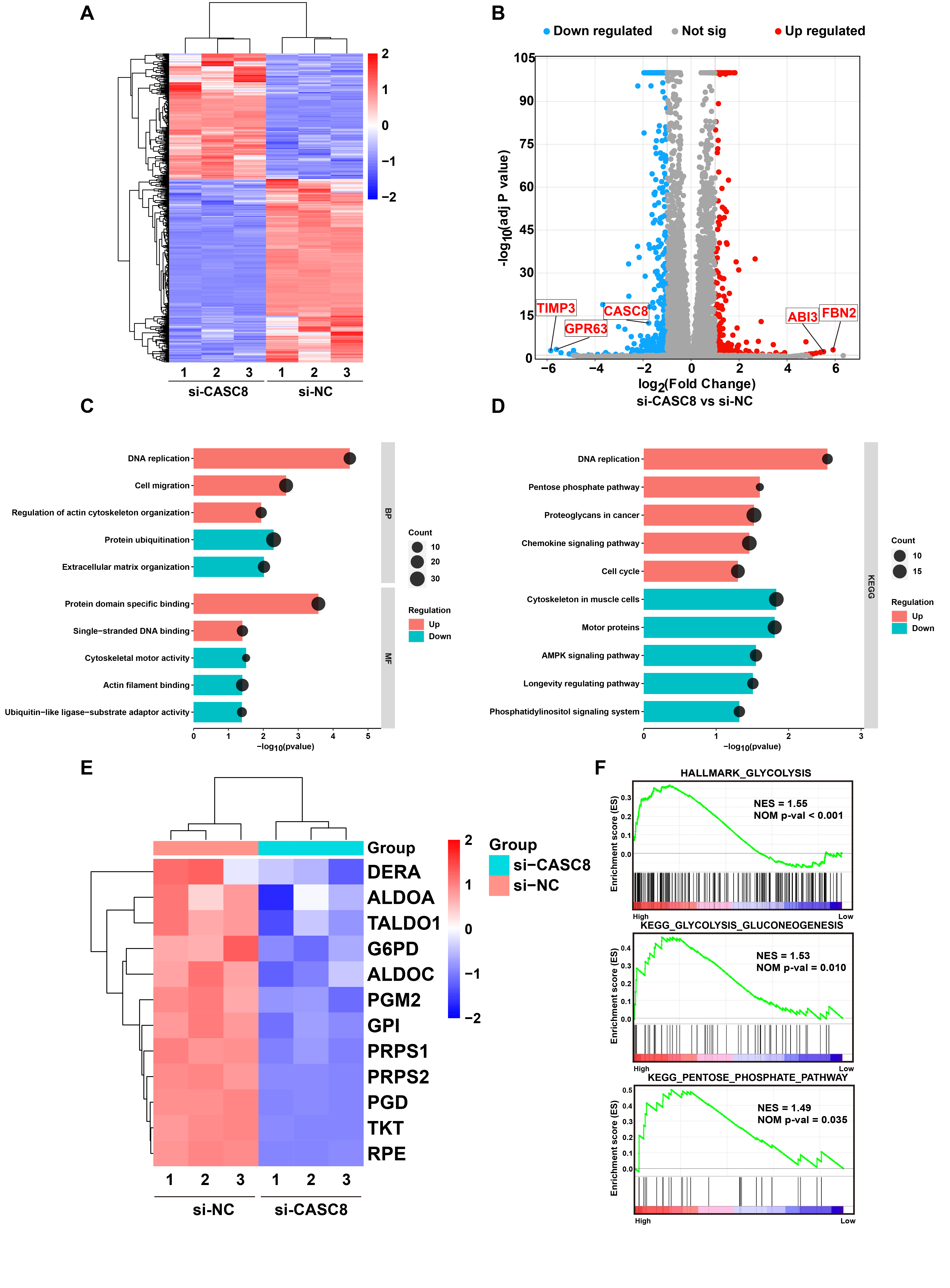

Supplement: Supplementary file 5 — Supplementary Material 5: Supplementary Fig. 5. The enrichment analysis of the DEGs between the control cells and CASC8 knockdown cells. (A) Gene expression heatmap of the control cells and CASC8 knockdown cells. (B) Volcano plotting of the control cells and CASC8 knockdown cells. The most significantly upregulated or downregulated genes and CASC8 were marked. (C) The results of GO enrichment analysis in the control group compared to CASC8 knockdown group. (D) The results of KEGG enrichment analysis in the control group compared to CASC8 knockdown group. (E) Gene expression heatmap of genes involved in phosphate pentose pathway between the control cells and CASC8 knockdown cells. (F) GSEA analysis between the control cells and CASC8 knockdown cells by using hallmark gene sets and KEGG gene sets. [file 13046_2025_3295_MOESM5_ESM.jpg]

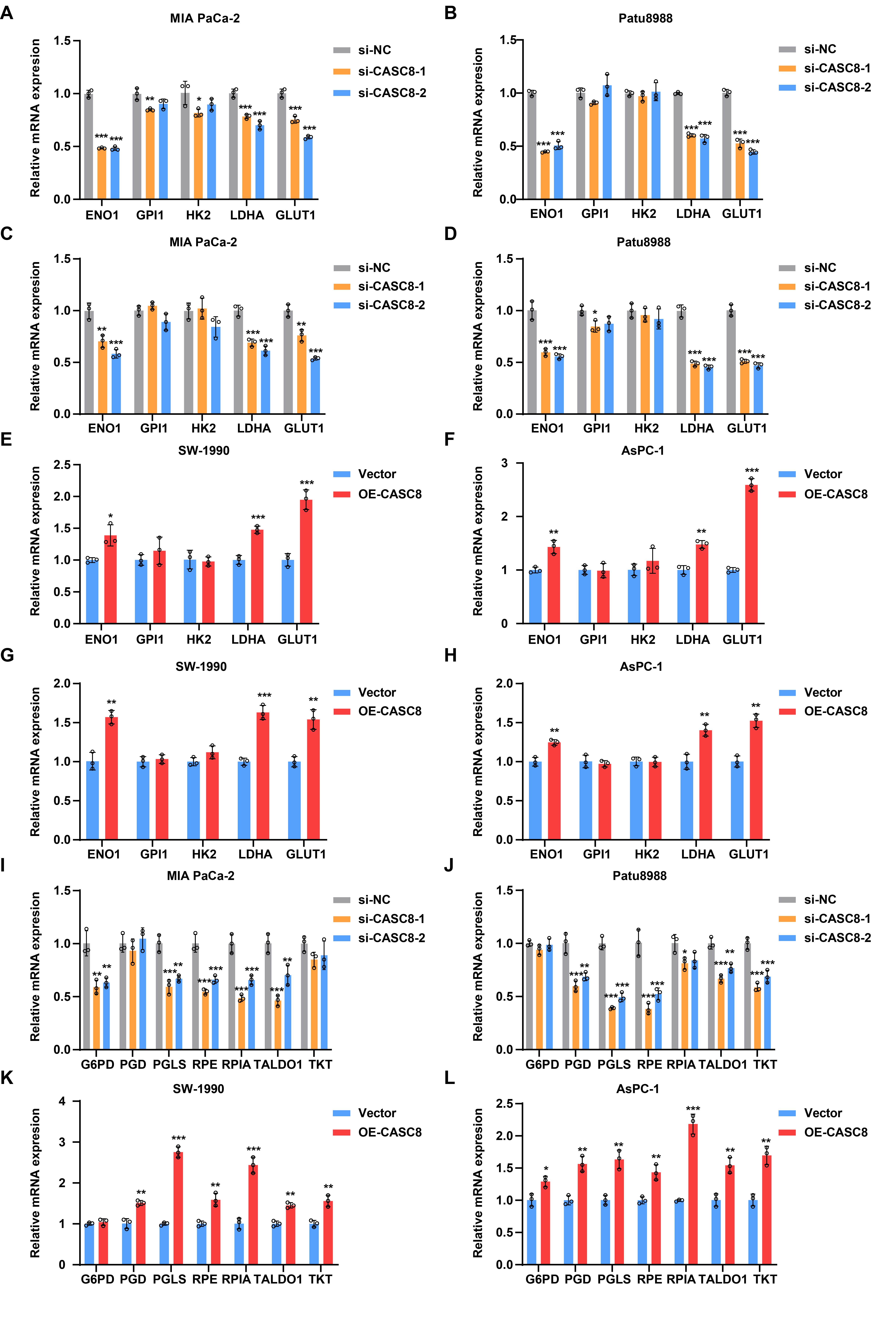

Supplement: Supplementary file 6 — Supplementary Material 6: Supplementary Fig. 6. CASC8 knockdown or overexpression modulates the expression of genes involved in glycolysis and pentose phosphate pathway. (A-B) qPCR analysis of glycolysis-related genes in MIA PaCa-2 (A) and Patu8988 (B) after CASC8 knockdown. Statistical significances were calculated by one-way ANOVA. (C-D) qPCR analysis of glycolysis-related genes in MIA PaCa-2 (C) and Patu8988 (D) following CASC8 knockdown under conditions of glucose starvation. Statistical significances were calculated by one-way ANOVA. (E–F) qPCR analysis of glycolysis-related genes in SW-1990 (E) and AsPC-1 (F) after CASC8 overexpression. Statistical significances were calculated by unpaired t-test. (G-H) qPCR analysis of glycolysis-related genes in SW-1990 (G) and AsPC-1 (H) following CASC8 overexpression under conditions of glucose starvation. Statistical significances were calculated by unpaired t-test. (I-J) qPCR analysis of genes involved in phosphate pentose pathway in MIA PaCa-2 (I) and Patu8988 (J) following CASC8 knockdown under conditions of glucose starvation. Statistical significances were calculated by one-way ANOVA. (K-L) qPCR analysis of genes involved in phosphate pentose pathway in SW-1990 (K) and AsPC-1 (L) following CASC8 overexpression under conditions of glucose starvation. Statistical significances were calculated by unpaired t-test. *** P < 0.001; ** P < 0.01; * P < 0.05. [file 13046_2025_3295_MOESM6_ESM.jpg]

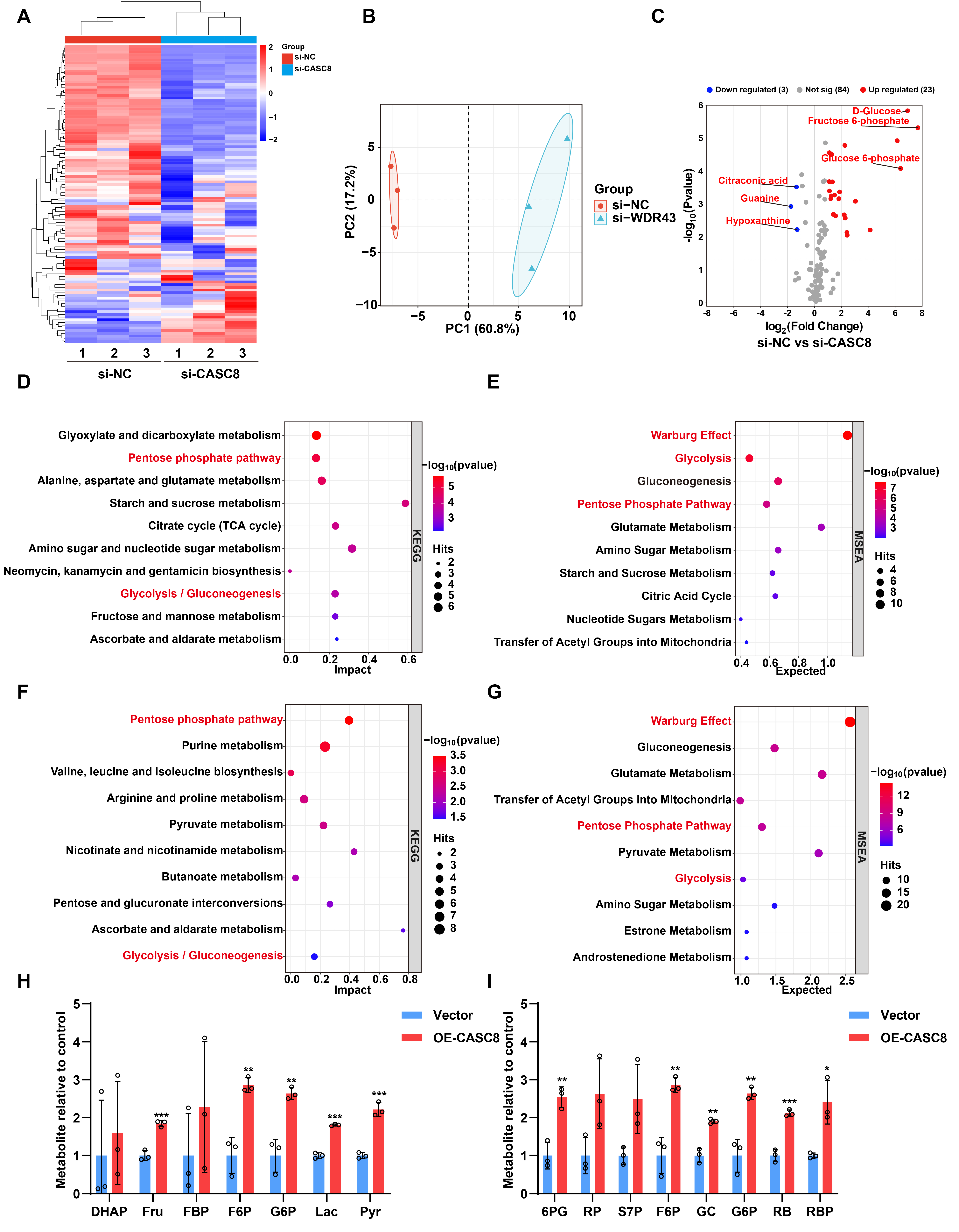

Supplement: Supplementary file 7 — Supplementary Material 7: Supplementary Fig. 7. Metabolomics analysis identified the effects of CASC8 on cellular metabolism. (A) Metabolites levels heatmap of the control and CASC8 knockdown MIA PaCa-2 cells under glucose starvation conditions. (B) Results of the principal component analysis (PCA) for the control cells and CASC8 knockdown cells. (C) Volcano plot of differential metabolites between control cells and CASC8 knockdown cells. The most significantly upregulated or downregulated metabolites were marked. (D) The KEGG enrichment analysis of differential metabolites upregulated in the control cells. (E) The MSEA enrichment analysis of differential metabolites upregulated in the control cells. (F) The KEGG enrichment analysis of differential metabolites upregulated in the CASC8 overexpression cells. (G) The MSEA enrichment analysis of differential metabolites upregulated in the CASC8 overexpression cells. (H-I) The levels of glycolysis metabolites (H) and the intermediates of phosphate pentose pathway (I) in SW-1990 with CASC8 overexpression under glucose starvation conditions. All metabolite levels were normalized to the vector cells. Statistical significances were calculated by unpaired t-test. DHAP, dihydroxyacetone phosphate; Fru, fructose; FBP, fructose 1,6-bisphosphate; F6P, fructose 6-phosphate; G6P, glucose 6-phosphate; Lac, lactate; Pyr, pyruvate; 6PG, 6-phosphogluconate; RP, D-ribulose 5-phosphate; S7P, D-sedoheptulose 7-phosphate; GC, gluconic acid; RB, ribose; RBP, ribose 1,5-bisphosphate. *** P < 0.001; ** P < 0.01; * P < 0.05. [file 13046_2025_3295_MOESM7_ESM.jpg]

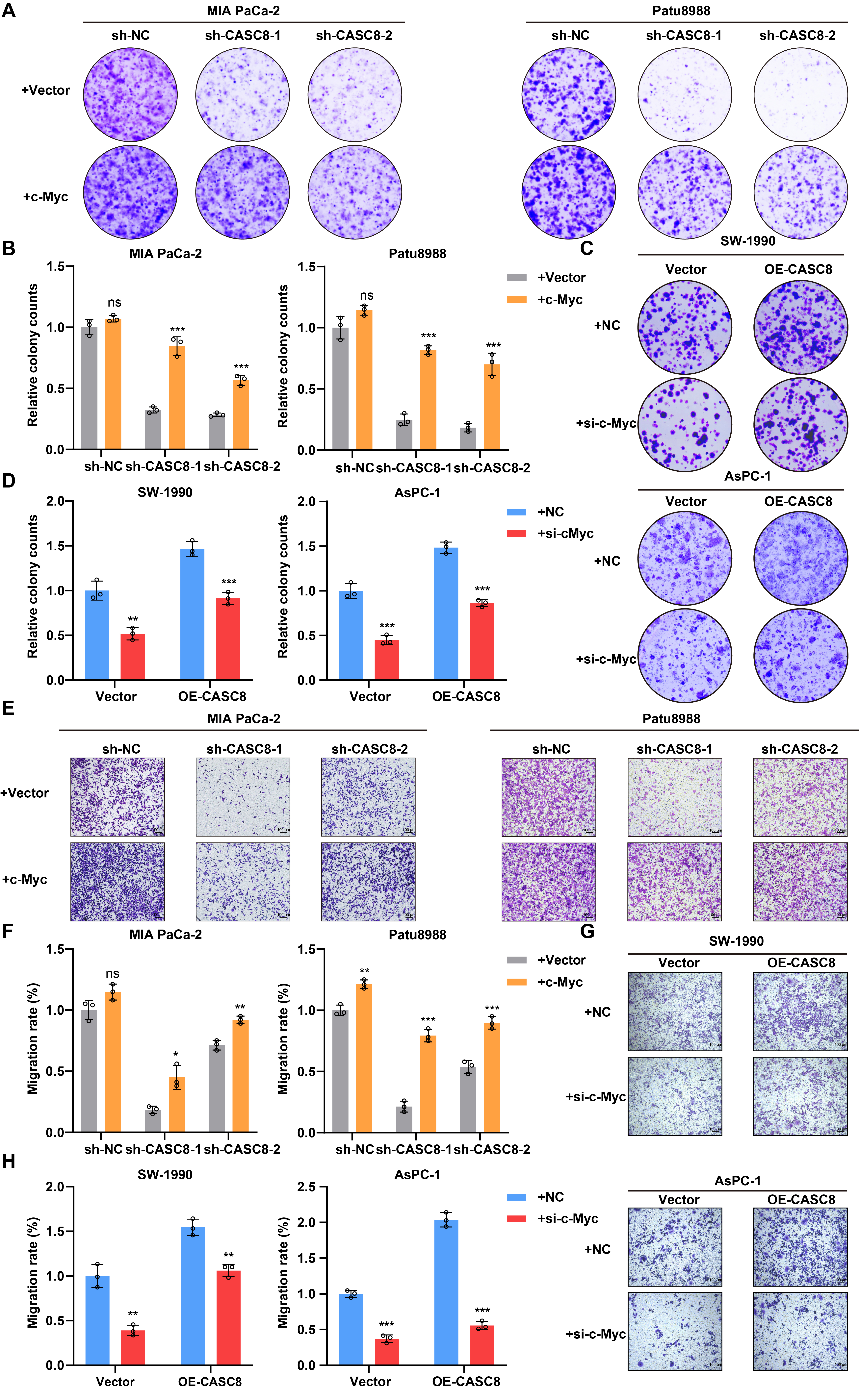

Supplement: Supplementary file 8 — Supplementary Material 8: Supplementary Fig. 8. CASC8 promotes PDAC growth and migration by regulating c-Myc. (A-B) Colony-formation assay (A) and statistical analysis (B) of CASC8 knockdown cells after c-Myc overexpression. (C-D) Colony-formation assay (C) and statistical analysis (D) of CASC8 overexpression cells after c-Myc knockdown. (E–F) Cell migration assay (E) and statistical analysis (F) of CASC8 knockdown cells after c-Myc overexpression. (G-H) Cell migration assay (G) and statistical analysis (H) of CASC8 overexpression cells after c-Myc knockdown. Scale bar = 100 μm. Statistical significances were calculated by unpaired t-test. *** P < 0.001; ** P < 0.01; * P < 0.05. [file 13046_2025_3295_MOESM8_ESM.jpg]

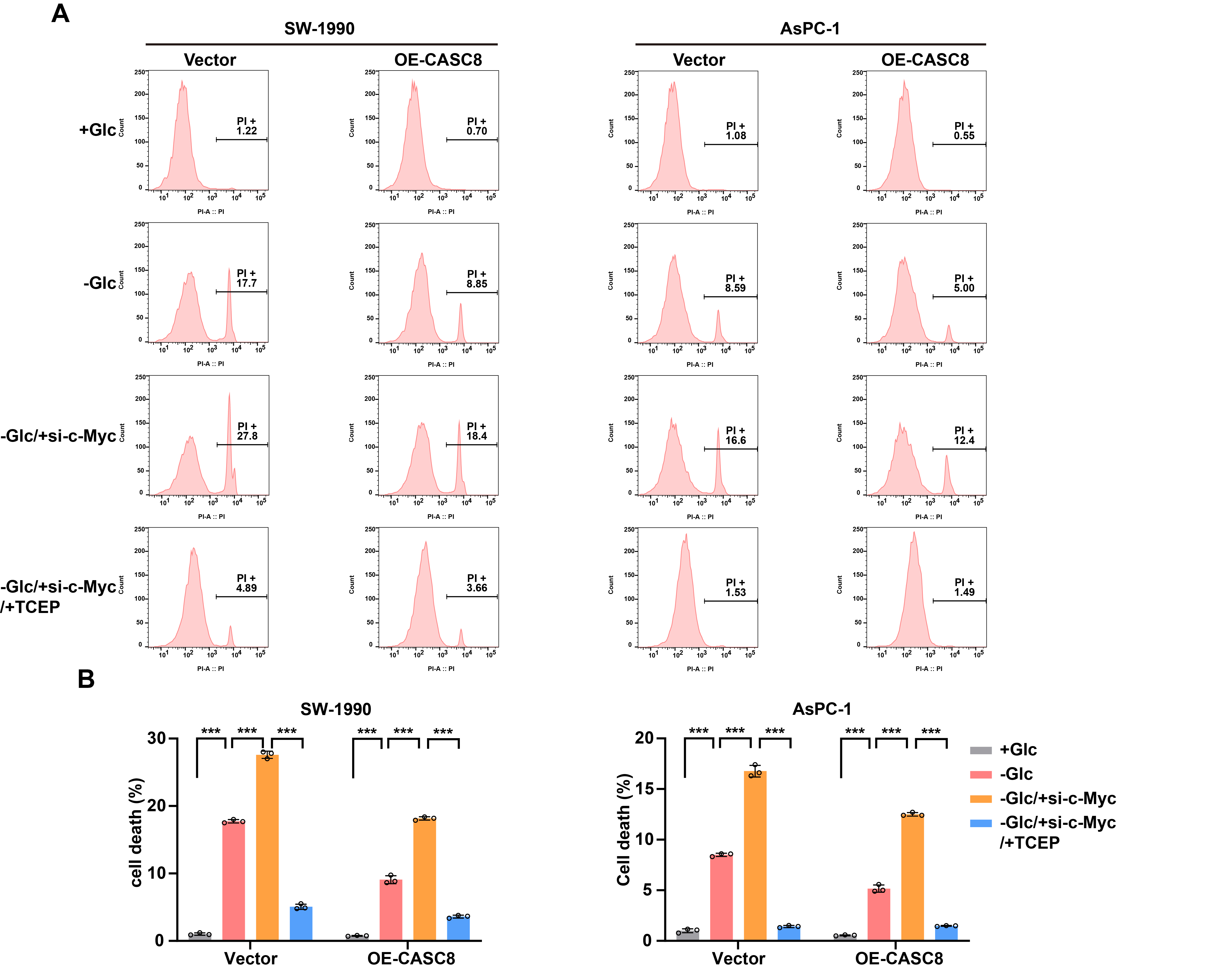

Supplement: Supplementary file 9 — Supplementary Material 9: Supplementary Fig. 9. CASC8 inhibits PDAC cells disulfidptosis via c-Myc. (A) The effects of c-Myc knockdown in CASC8 overexpression cells on cell death after cultured in glucose-free medium for 12 h and with or without 1 mM TCEP. (B) Statistical analysis of the PI-positive cells in SW-1990 and AsPC-1 after CASC8 overexpression and following c-Myc knockdown. P -values between the two interested groups were calculated by unpaired t-test. *** P < 0.001; ** P < 0.01; * P < 0.05. [file 13046_2025_3295_MOESM9_ESM.jpg]
