# Supplementary material for: CASC8 activates the pentose phosphate pathway to inhibit disulfidptosis in pancreatic ductal adenocarcinoma though the c-Myc-GLUT1 axis
Source: J Exp Clin Cancer Res. 2025 Jan 27;44:26. doi: 10.1186/s13046-025-03295-w (PMC11771065; doi:10.1186/s13046-025-03295-w)
Supplement: Supplementary file 10 — Supplementary Material 10: Supplementary Table. The primers used in this study. [file 13046_2025_3295_MOESM10_ESM.docx]

**Supplementary Table. The primers used in this study**

| **Gene** | **Primers sequences** | **Amplicon size** | **Accession no.** |
| --- | --- | --- | --- |
| DDX18 forward  DDX18 reverse | 5′-ATGTCACACCTGCCGATGAAA-3′  5′-CCCTGAAACTTTAGGTTCCGC-3′ | 86 | [NM_006773](http://www.ncbi.nlm.nih.gov/entrez/query.fcgi?cmd=Search&db=Nucleotide&term=NM_006773) |
| GNL3 forward  GNL3 reverse | 5′-ATGACCTGCCATAAGCGGTAT-3′  5′-CTTAAAGGGAGCACTGTTTGGA-3′ | 135 | [[NM_206825](http://www.ncbi.nlm.nih.gov/entrez/query.fcgi?cmd=Search&db=Nucleotide&term=NM_206825)4](http://www.ncbi.nlm.nih.gov/entrez/query.fcgi?cmd=Search&db=Nucleotide&term=NM_033294) |
| HSPD1 forward  HSPD1 reverse | 5′-ATGCTTCGGTTACCCACAGTC-3′  5′-AGCCCGAGTGAGATGAGGAG-3′ | 75 | [NM_002156](http://www.ncbi.nlm.nih.gov/entrez/query.fcgi?cmd=Search&db=Nucleotide&term=NM_002156) |
| MCM4 forward  MCM4 reverse | 5′-GACGTAGAGGCGAGGATTCC-3′  5′-GCTGGGAGTGCCGTATGTC-3′ | 182 | [NM_005914](http://www.ncbi.nlm.nih.gov/entrez/query.fcgi?cmd=Search&db=Nucleotide&term=NM_005914) |
| NOP16 forward  NOP16 reverse | 5′-GGTTACAGTGTCAACCGAAAGC-3′  5′-GATGTGGGAGCATTCGATCCG-3′ | 78 | [NM_001256540](http://www.ncbi.nlm.nih.gov/entrez/query.fcgi?cmd=Search&db=Nucleotide&term=NM_001256540) |
| [NPM1](http://www.ncbi.nlm.nih.gov/entrez/query.fcgi?cmd=Search&db=Nucleotide&term=NM_001256540) forward | 5′-GGAGGTGGTAGCAAGGTTCC-3′ | 143 | [NM_002520](http://www.ncbi.nlm.nih.gov/entrez/query.fcgi?cmd=Search&db=Nucleotide&term=NM_002520) |
| NPM1 reverse | 5′-TTCACTGGCGCTTTTTCTTCA-3′ |  |  |
| PA2G4 forward | 5′-CAGGAGCAAACTATCGCTGAG-3′ | 81 | [NM_006191](http://www.ncbi.nlm.nih.gov/entrez/query.fcgi?cmd=Search&db=Nucleotide&term=NM_006191) |
| PA2G4 reverse | 5′-GGACCGAAGTACCCTGTTGG-3′ |  |  |
| MYC forward | 5′-GGCTCCTGGCAAAAGGTCA-3′ | 119 | [NM_002467](http://www.ncbi.nlm.nih.gov/entrez/query.fcgi?cmd=Search&db=Nucleotide&term=NM_002467) |
| MYC reverse | 5′-CTGCGTAGTTGTGCTGATGT-3′ |  |  |
| G6PD forward | 5′-CGAGGCCGTCACCAAGAAC-3′ | 166 | [NM_000402](http://www.ncbi.nlm.nih.gov/entrez/query.fcgi?cmd=Search&db=Nucleotide&term=NM_000402) |
| G6PD reverse | 5′-GTAGTGGTCGATGCGGTAGA-3′ |  |  |
| PGD forward | 5′-ATGGCCCAAGCTGACATCG-3′ | 81 | [NM_002631](http://www.ncbi.nlm.nih.gov/entrez/query.fcgi?cmd=Search&db=Nucleotide&term=NM_002631) |
| PGD reverse | 5′-AAAGCCGTGGTCATTCATGTT-3′ |  |  |
| PGLS forward | 5′-GGAGCCTCGTCTCGATGCTA-3′ | 160 | [NM_012088](http://www.ncbi.nlm.nih.gov/entrez/query.fcgi?cmd=Search&db=Nucleotide&term=NM_012088) |
| PGLS reverse | 5′-GAGAGAAGATGCGTCCGGT-3′ |  |  |
| RPE forward | 5′-AAGCCAGAACAGTGGGTAAAGC-3′ | 184 | [NM_006916](http://www.ncbi.nlm.nih.gov/entrez/query.fcgi?cmd=Search&db=Nucleotide&term=NM_006916) |
| RPE reverse | 5′-CAAGGCCAACCTGCAATGG-3′ |  |  |
| RPIA forward | 5′-AGTGCTGGGAATTGGAAGTGG-3′ | 97 | [NM_144563](http://www.ncbi.nlm.nih.gov/entrez/query.fcgi?cmd=Search&db=Nucleotide&term=NM_144563) |
| RPIA reverse | 5′-GGGAATACAGACGAGGTTCAGA-3′ |  |  |
| TALDO1 forward | 5′-CTCACCCGTGAAGCGTCAG-3′ | 127 | [NM_006755](http://www.ncbi.nlm.nih.gov/entrez/query.fcgi?cmd=Search&db=Nucleotide&term=NM_006755) |
| TALDO1 reverse | 5′-GTTGGTGGTAGCATCCTGGG-3′ |  |  |
| TKT forward | 5′-TCCACACCATGCGCTACAAG-3′ | 164 | [NM_001064](http://www.ncbi.nlm.nih.gov/entrez/query.fcgi?cmd=Search&db=Nucleotide&term=NM_001064) |
| TKT reverse | 5′-CAAGTCGGAGCTGATCTTCCT-3′ |  |  |
| ENO1 forward | 5′-AAAGCTGGTGCCGTTGAGAA-3′ | 217 | [NM_001201483](http://www.ncbi.nlm.nih.gov/entrez/query.fcgi?cmd=Search&db=Nucleotide&term=NM_001201483) |
| ENO1 reverse | 5′-GGTTGTGGTAAACCTCTGCTC-3′ |  |  |
| GPI forward | 5′-CAAGGACCGCTTCAACCACTT-3′ | 239 | [NM_001184722](http://www.ncbi.nlm.nih.gov/entrez/query.fcgi?cmd=Search&db=Nucleotide&term=NM_001184722) |
| GPI reverse | 5′-CCAGGATGGGTGTGTTTGACC-3′ |  |  |
| HK2 forward | 5′-GAGCCACCACTCACCCTACT-3′ | 249 | [NM_000189](http://www.ncbi.nlm.nih.gov/entrez/query.fcgi?cmd=Search&db=Nucleotide&term=NM_000189) |
| HK2 reverse | 5′-CCAGGCATTCGGCAATGTG-3′ |  |  |
| LDHA forward | 5′-ATGGCAACTCTAAAGGATCAGC-3′ | 86 | [NM_001165415](http://www.ncbi.nlm.nih.gov/entrez/query.fcgi?cmd=Search&db=Nucleotide&term=NM_001165415) |
| LDHA reverse | 5′-CCAACCCCAACAACTGTAATCT-3′ |  |  |
| GLUT1 forward | 5′-GGCCAAGAGTGTGCTAAAGAA-3′ | 201 | [NM_006516](http://www.ncbi.nlm.nih.gov/entrez/query.fcgi?cmd=Search&db=Nucleotide&term=NM_006516) |
| GLUT1 reverse | 5′-ACAGCGTTGATGCCAGACAG-3′ |  |  |
| SLC7A11 forward | 5′-TCTCCAAAGGAGGTTACCTGC-3′ | 123 | [NM_014331](http://www.ncbi.nlm.nih.gov/entrez/query.fcgi?cmd=Search&db=Nucleotide&term=NM_014331) |
| SLC7A11 reverse | 5′-AGACTCCCCTCAGTAAAGTGAC-3′ |  |  |
| 18S forward | 5′-ATCACCATTATGCAGAATCCACG-3 | 93 | [NM_022551](http://www.ncbi.nlm.nih.gov/entrez/query.fcgi?cmd=Search&db=Nucleotide&term=NM_022551) |
| 18S reverse | 5′-GACCTGGCTGTATTTTCCATCC-3′ |  |  |
